# Transcriptome analysis of the role of autophagy in plant response to heat stress

**DOI:** 10.1371/journal.pone.0247783

**Published:** 2021-02-26

**Authors:** Yan Zhang, Haoxuan Min, Chengchen Shi, Gengshou Xia, Zhibing Lai

**Affiliations:** 1 Department of Landscape and Horticulture, Ecology College, Lishui University, Lishui, Zhejiang, China; 2 National Key Laboratory of Crop Genetic Improvement, Huazhong Agricultural University, Wuhan, China; CSIR- Institute of Himalayan Bioresource Technology, INDIA

## Abstract

Autophagy plays a critical role in plant heat tolerance in part by targeting heat-induced nonnative proteins for degradation. Autophagy also regulates metabolism, signaling and other processes and it is less understood how the broad function of autophagy affects plant heat stress responses. To address this issue, we performed transcriptome profiling of Arabidopsis wild-type and autophagy-deficient *atg5* mutant in response to heat stress. A large number of differentially expressed genes (DEGs) were identified between wild-type and *atg5* mutant even under normal conditions. These DEGs are involved not only in metabolism, hormone signaling, stress responses but also in regulation of nucleotide processing and DNA repair. Intriguingly, we found that heat treatment resulted in more robust changes in gene expression in wild-type than in the *atg5* mutant plants. The dampening effect of autophagy deficiency on heat-regulated gene expression was associated with already altered expression of many heat-regulated DEGs prior to heat stress in the *atg5* mutant. Altered expression of a large number of genes involved in metabolism and signaling in the autophagy mutant prior to heat stress may affect plant response to heat stress. Furthermore, autophagy played a positive role in the expression of defense- and stress-related genes during the early stage of heat stress responses but had little effect on heat-induced expression of heat shock genes. Taken together, these results indicate that the broad role of autophagy in metabolism, cellular homeostasis and other processes can also potentially affect plant heat stress responses and heat tolerance.

## Introduction

Heat is a common stress for all organisms, particularly for sessile organisms like plants [1]. Global warming associated with climate change leads to more extremely high temperature, which will impact plant growth, seriously limit crop productivity and threaten food security. Heat is a fundamental threat to all forms of life and, therefore, responses to heat stress characterized by rapid expression of genes encoding heat shock proteins (HSPs) have evolved universally. Many of these HSPs are protein chaperones that promote the folding and refolding of nonnative proteins that are produced under high temperature [2–6]. In addition, plants have evolved other complex tolerance mechanisms that employ ion transporters, osmoprotectants, antioxidants, plant hormones and other factors to offset heat-induced biochemical and physiological alterations and mitigate their harmful effects [1, 7, 8]. More recent studies have also established a critical role of protein quality control processes such as autophagy in plant responses to heat stress [9–11].

Autophagy is a highly conserved process that degrades and recycles intracellular components including damaged proteins and organelles [12–14]. Central to autophagy is the formation of autophagosomes. More than 40 autophagy-related (ATG) proteins in yeast cooperate to perform the physiologically continuous but mechanistically distinct processes of autophagy including the induction of autophagy, autophagosome nucleation, elongation, maturation, and fusion with the vacuole [12, 15, 16]. The core process of autophagy and ATG proteins are highly conserved in all eukaryotic organisms including plants. Genetic and molecular studies over the past two decades or so have established an important role of autophagy in almost all aspects of plant life, particularly in plant responses to various stresses conditions [17–20]. Autophagy and ATG gene expression are induced by diverse abiotic stress conditions including nutrient starvation, heat, salt, drought and oxidative stresses [9–11, 21–25]. *A*utophagy mutants and transgenic silencing lines are all hypersensitive to or compromised in tolerance to these abiotic stresses [9–11, 21–25]. Autophagy also plays an important role in plant innate immunity based on altered phenotypes of plant mutants or transgenic silencing lines in responses to virulent and avirulent biotrophic pathogens, necrotrophic and viral pathogens [26–34]. Autophagy also plays important roles in root growth, leaf senescence, pollen and endosperm development [35, 36].

Even though the nonselective process of bulk degradation of intracellular contents is a major mode of action by autophagy in nutrient recycling, the broad roles of autophagy are primarily mediated by selective clearance of specific cellular structures through the action of selective autophagy receptors [37–40]. For example, selective autophagy targets degradation of misfolded and other nonnative proteins generated under high temperature and, therefore, plays an important role in plant response to heat stresses [9, 10, 25, 41]. Plant NBR1, a structural homolog and functional hybrid of mammalian autophagy receptors NBR1 and p62, plays an important role in plant heat tolerance [9, 10]. NBR1 interacts with autophagosome-anchored ATG8 proteins through its ATG8-interacting motifs and recognize its ubiquitinated protein aggregates through its ubiquitin-associated domains (UBAs), thereby recruiting its cargo into the autophagosome for eventual degradation in the vacuole [9, 11]. Arabidopsis mutants and transgenic silencing tomato plants for NBR1 displayed compromised heat tolerance, which was associated with elevated accumulation of insoluble, detergent-resistant protein aggregates that were also highly ubiquitinated under heat stress [9, 11]. More recent research has further demonstrated that NBR1-mediated selective autophagy plays a critical role in plant response to recurrent heat stress by targeting degradation of HSP90.1 and its interacting partner ROF1 (rotamase FKBP 1). Formation of the HSP90.1-ROF1 complex is probably required for enhanced transcriptional activity of heat shock transcription factor HSFA2 and sustained HSP synthesis during heat stress recovery to improve plant response to an imminent recurrence of heat stress [42]. In addition, the three related ATG8-interacting 3 (ATI3) proteins also function as selective autophagy receptors with a critical role in plant heat tolerance by interacting with endoplasmic reticulum(ER)-localized UBAC2 proteins and mediating selective autophagy of specific unknown ER components [25].

Selective degradation of heat-induced nonnative proteins is an important mechanism by which autophagy promotes plant heat tolerance. However, autophagy also regulates other cellular processes including metabolism, protein homeostasis and signaling [17, 43]. However, there has been no reported study using a comprehensive genomic approach to address whether the broad function of autophagy can also affect plant heat stress responses and heat tolerance. To gain insights into the broad function of autophagy in plant heat tolerance, we performed comparative transcriptome profiling of Arabidopsis wild-type and autophagy-deficient *atg5* mutant. We observed major transcriptomic alterations between wild-type and *atg5* mutant even under normal growth conditions and identified a large number of differential expressed genes (DEGs) involved not only in metabolism, hormone signaling, stress responses but also in regulation of cell cycle, nucleotide processing and DNA repair. Heat treatment led to more robust changes in gene expression in wild-type plants than in the *atg5* mutant, largely due to the fact that expression of many heat-regulated DEGs were already altered in the *atg5* mutant prior to heat stress. The DEGs co-regulated by heat and autophagy included those involved in metabolism, stress responses and hormone signaling and likely have a critical role in plant heat tolerance. Furthermore, autophagy positively modulated the expression of defense- and stress-related genes during the early stage of heat stress responses but heat induction of heat shock protein genes was not significantly affected in the *atg5* mutant. Collectively, these results revealed potential new mechanisms by which autophagy affects plant heat tolerance through its broad function in regulation of metabolism and cellular homeostasis.

## Materials and methods

### Plant materials and growth condition

The Arabidopsis plants used in this study are in the Col-0 background. The autophagy deficient) *atg5* mutant (SAIL_129_B07) has been previously described [29]. Arabidopsis plants were grown in a growth chamber at 22±2°C under 200 μmol m^-2^ s^-1^ light with a 12 h light/12 h dark cycle. For heat treatment, five-week-old plants were placed into growth chamber at 45°C under 200 μmol m^-2^ s^-1^ with the same light/dark cycle.

### Total RNA isolation, library construction and RNA-seq

Total RNA was isolated from 5-week-old plants using Trizol reagent (Sigma, USA), according to the manufacturer’s instructions. Genomic DNA was removed with DNaseⅠ (RNase-free) (NEB, USA). RNA purity, concentration and integrity were confirmed using Nanodrop (Thermo Scientific, USA), Qubit 2.0 fluorometer (Thermo Scientific, USA) and Aglient 2100 Bioanalyzer (Agilent, USA), respectively.

For construction of cDNA library, mRNA was isolated from total RNA using poly(dT) oligo-attached magnetic beads. First-strand cDNA was synthesized from fragmented mRNA using random primers. Second strand cDNA was synthesized using RNase H and DNA polymerase I. Double-strand cDNA were purified by AMPure XP beads and the cDNA library was constructed after PCR enrichment. Two biological replicates were used for every time point of heat treatment. Samples were subjected to high throughput sequencing using Illumina HiSeq2500.

### Quantification of transcripts and identification of differentially expressed genes

The adapter sequences from the RNA-seq data were trimmed and low-quality reads were removed from raw data to gain clean reads. Clean reads were aligned to Arabidopsis genome TAIR10 [44] to obtain and improve mapped reads using TopHat2 [45] and Bowtie [46] softwares, respectively. Alternative Splicing Events were predicted according to trans-intron reads compared with known splicing model by Cufflinks [47]. BLAST [48] was used to obtain annotations for new genes using data bank NR [49], Swiss-Prot [50], GO [51], COG [52], KOG [53], Pfam [54] and KEGG [54].

Read Per Kilobase of transcript per Million fragments mapped (RPKM) [55] was calculated by Cuffdiff to measure gene expression quantity. Fold-Change≥2, Fold-Change ≤0.5 and False Discovery Rate (FDR) <0.01 were used as standard to identify Differentially Expressed Gene (DEG) by DESeq [54]. Pearson’s Correlation Coefficient (r^2^) was used as evaluation index for biological replicates.

### Bioinformatic analyses

Principal Component Analysis (PCA) was performed using R studio with ggplot2 package. AgriGO was used to determine gene ontology (GO) enrichment [56]. The ShinoGO v0.61 web tool (http://bioinformatics.sdstate.edu/go/) was used to identify KEGG pathways [57].

## Results

### Principal Component Analyses of transcriptome data

**Fig 1:**
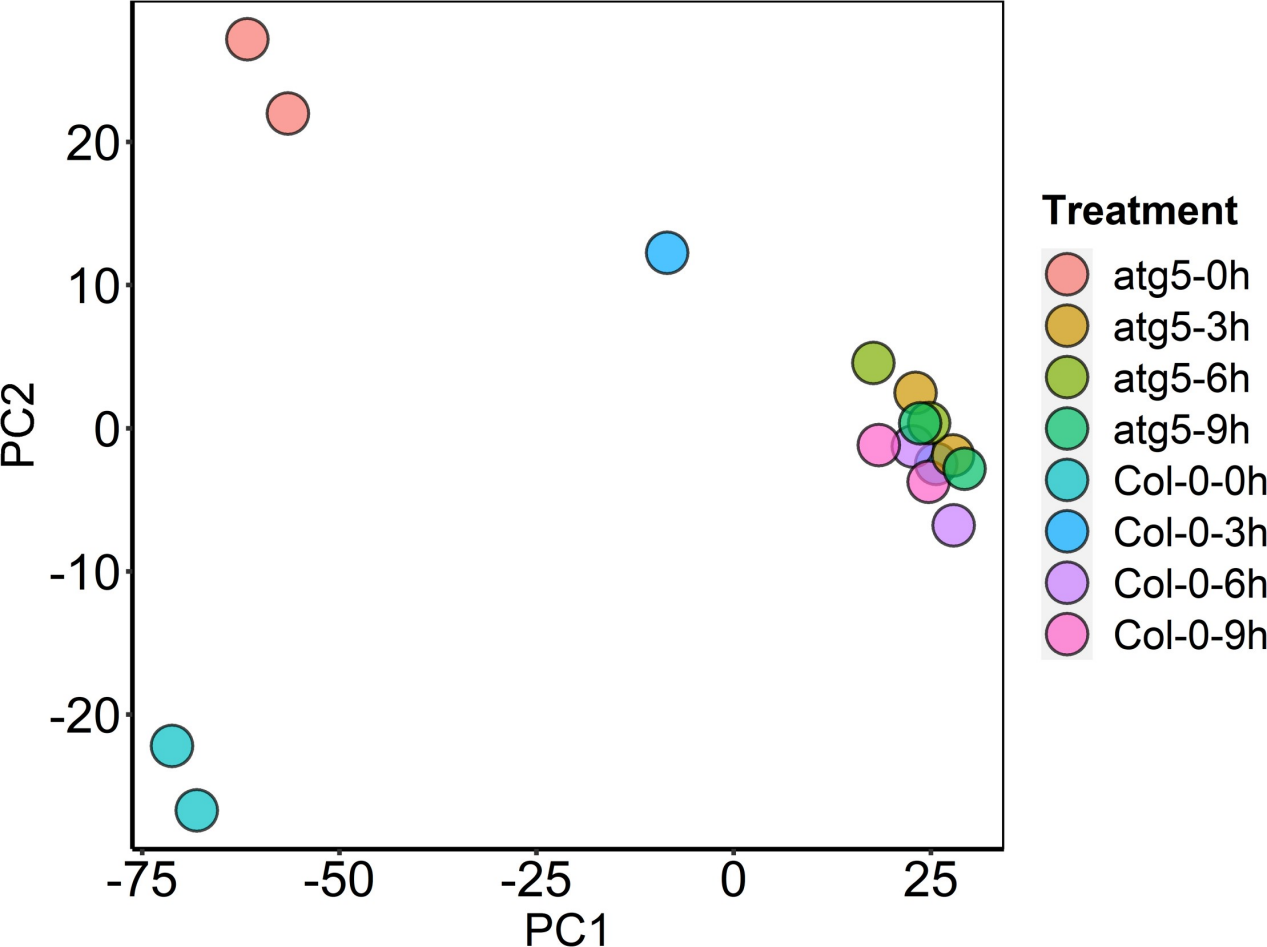
Principal Component (PC) analysis. The plot displays all 16 samples along PC1 and PC2, which account for 79% and 9% of the variability, respectively, within the expression data set. PC analysis was applied to normalized RPKM and log-transformed count data.

To perform transcriptome profiling for assessing the effects of autophagy on plant heat stress responses, we subjected Col-0 wild-type and autophagy-deficient *atg5* plants to heat treatment for 0, 3, 6, 9 hours and sampled rosette leaves for total RNA isolation and sequencing. The transcriptome data of the sixteen samples (T1-T16) described in the study haven been deposited into NCBI databases and the bioproject accession number is PRJNA548241. From sixteen samples (two genotypes, four time points and two biological replicates), we obtained 348 million total filtered reads, of which 174.19 million could be mapped to the Arabidopsis genome assembly, with 17.1 to 20.9 million uniquely mapped reads per sample (S1 Fig). To obtain the normalized expression values, we calculated RPKMs for all genes in the samples to exhibit distribution of total genes expression (S2 Fig). Pearson’s Correlation Coefficient (r^2^) analysis confirmed strong correlation between the biological replicates, indicating the high reproducibility of the transcriptome data (S1 Table). For further quality assessment and exploratory analysis of the transcriptome data, we performed Principal Component Analysis (PCA). As shown in Fig 1, the most variation within the data (PC1) accounted for 79% of the variance, which was primarily caused by variance between untreated and heat-treated samples. Notably, there was very limited variance among samples that had been subjected to 3, 6 and 9 hours of heat stress (Fig 1). PC2 accounted for an additional 9% of variance, primarily between untreated Col-0 and *atg5* mutant plants (Fig 1). Interestingly, there was relatively limited variance among heat-treated twelve samples of Col-0 and *atg5* mutant. However, even among the highly clustered heat-treated samples, there was significant variance between the heat-treated *atg5* and Col-0 samples (Fig 1). Taken together, PCA revealed a major difference in gene expression between untreated and heat-treated plants. Autophagy deficiency also has a substantial effect on gene expression but this effect was primarily on untreated plants.

### Differentially Expressed Gene (DEG) analysis between Col-0 and *atg5* mutant

**Fig 2:**
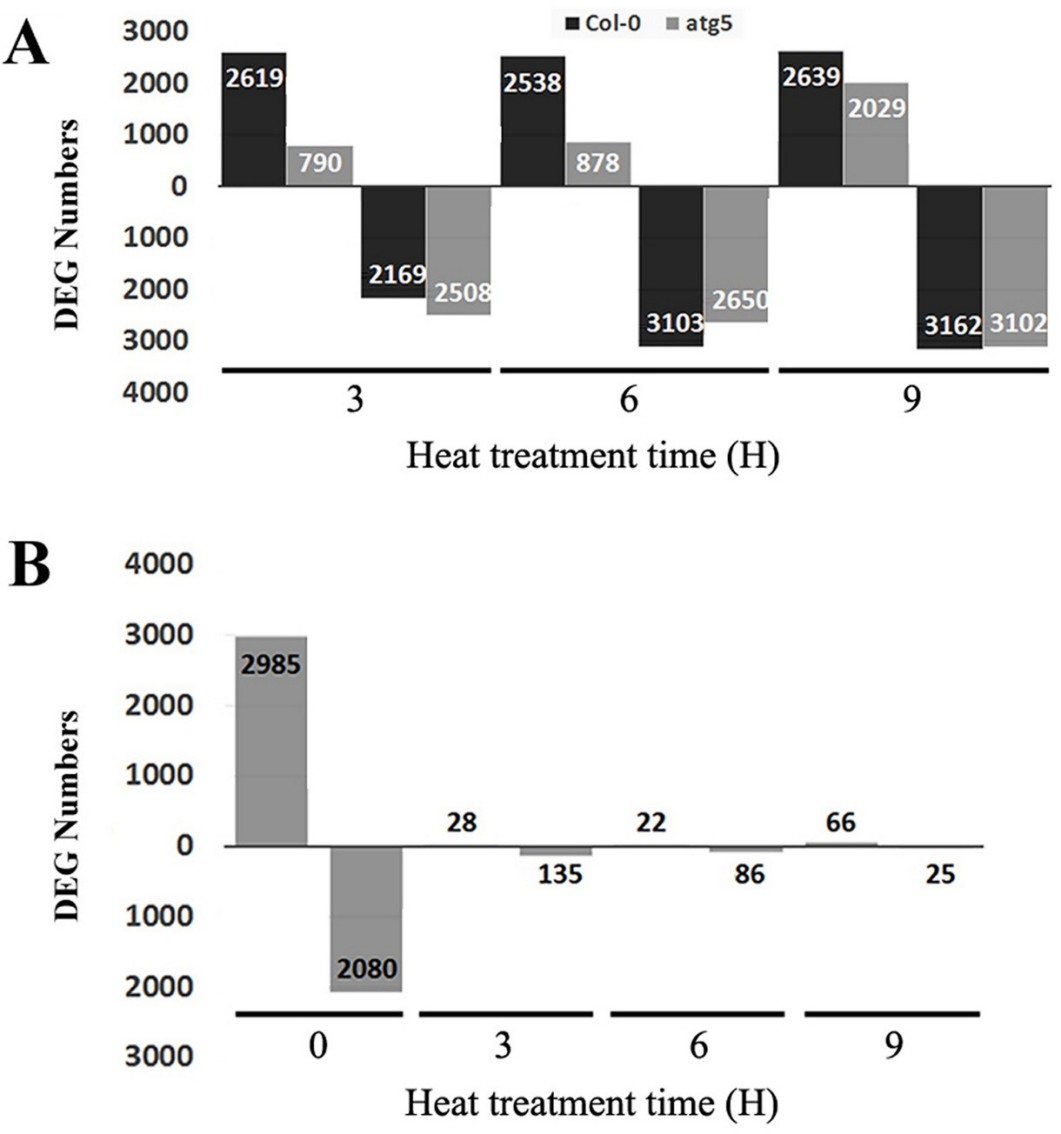
Differentially Expressed Genes (DEGs) identified from RNA-seq analysis in Col-0 and *atg5* mutant at 0, 3,6 and 9 hours of heat stress. A. Number of up- and down-regulated DEGs between heat-treated for 3, 6 or 9 hours vs untreated (0 hour of heat treatment) samples. B. Number of up- and down-regulated DEGs between Col-0 and *atg5* at indicated hours of heat treatment. Numbers of up-regulated (above X axis) and down-regulated (below X axis) DEGs are indicated.

In order to gain insights into the effects of heat stress and autophagy on gene expression, we analyzed differentially expressed genes (DEGs) from the transcriptome profiles. First, we analyzed Col-0 and *atg5* mutants independently for their respective DEGs through pairwise comparison of 3-, 6- or 9-hour heat-treated samples with the 0-hour heat-treated sample of the same genotype. As shown in Fig 2A, 3-hour heat treatment up-regulated more than 2600 genes in Col-0 wild-type plants but only 790 genes in *atg5* mutant. By contrast, 3-hour heat stress down-regulated similar numbers of genes (2169 and 2508, respectively) in Col-0 and *atg5* mutant plants (Fig 2A). Likewise, after 6-hour heat stress, there were approximately three times more genes up-regulated in Col-0 than in the *atg5* mutant plants (2538 and 878, respectively) (Fig 2A). Again, the numbers of down-regulated genes were more similar in Col-0 and *atg5* upon 6-hour heat exposure (Fig 2A). Interestingly, at 9-hour heat exposure, both up- and down-regulated DEG numbers were similar in Col-0 and *atg5* mutant plants (Fig 2A). Thus, at the relatively early stages of heat stress (3 and 6 hours at the high temperature), there was much more robust gene induction in Col-0 than in *atg5* mutant plants.

As shown in Fig 1, there was a much higher variance in the transcriptome data between Col-0 and *atg5* at 0 hour than at 3, 6 and 9 hours of heat treatment. To confirm and expand on this finding, we performed pairwise comparison between Col-0 and *atg5* to determine the numbers of DEGs at each of the four heat exposure times. As shown in Fig 2B, at 0 hour of heat treatment, there were 5065 DEGs with 2985 up-regulated and 2080 down-regulated between Col-0 and *atg5*. Therefore, gene expression profiles differed substantially between Col-0 and *atg5* even under normal growth temperature. Strikingly, the difference in the transcript profiles between Col-0 and *atg5* largely disappeared after heat stress based on the drastic reduction in the numbers of DEG at 3, 6 and 9 hours of heat stress. For example, the total numbers of up- and down-regulated DEGs at 3 hours of heat stress were only about 1 and 6% of those at 0 hour of heat stress (Fig 2B). A similar pattern was also observed for DEGs at 6 and 9 hours of heat stress (Fig 2B). These results further indicated heat stress greatly reduced the difference in gene expression profiles between Col-0 and *atg5* mutant plants.

### GO and KEGG analyses of DEGs between unstressed Col-0 and *atg5* mutant

**Fig 3:**
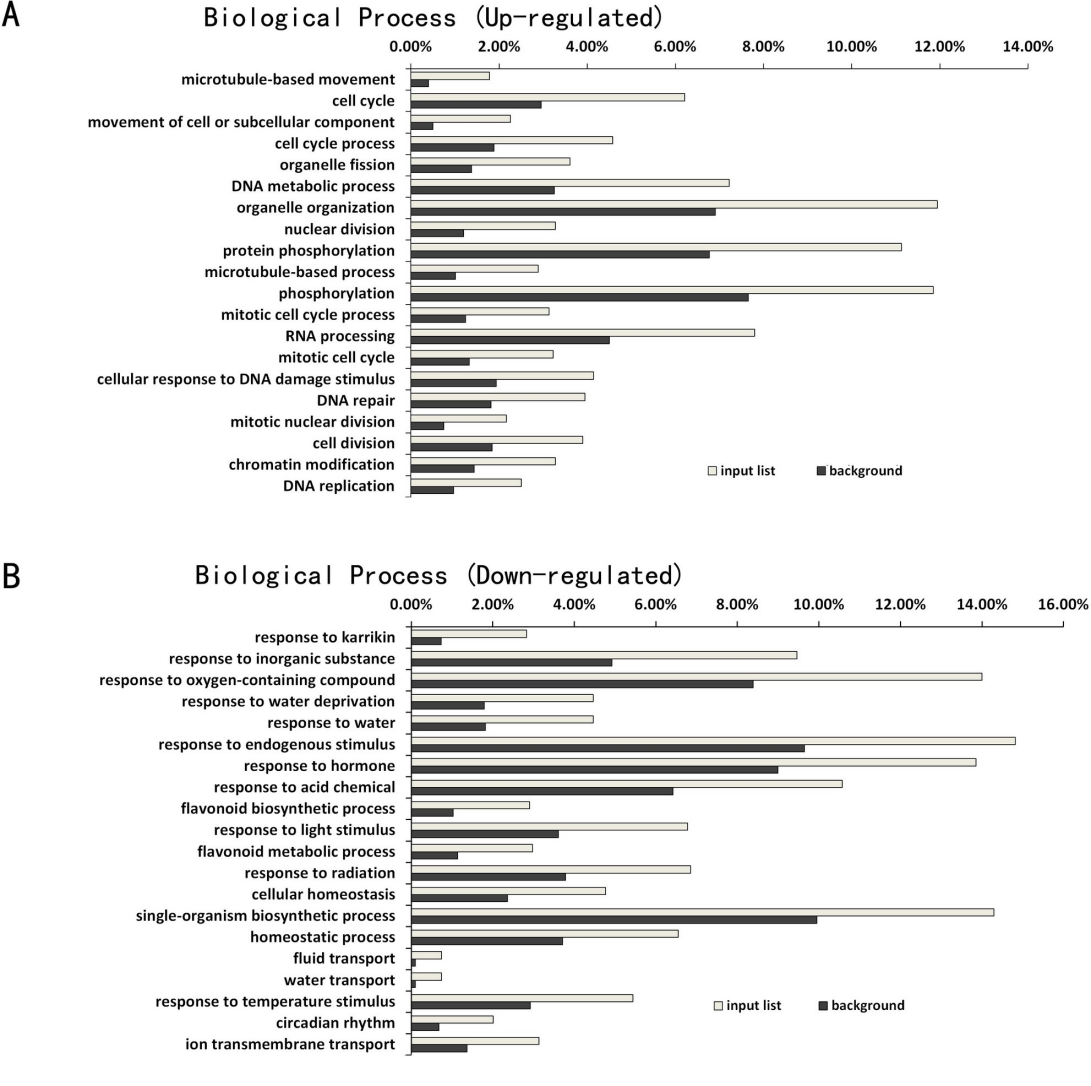
GO enrichment analysis of DEGs between Col-0 and *atg5* mutant at 0 hour of heat treatment. The enrichment analysis of up-regulated (A) and down-regulated (B) DEGs was performed using the GO term that describes biological process. The enrichment of DEGs for a specific biological process (input list) was calculated as percentage of the total number of DEGs identified in the RNA-seq data. For comparison, the percentage of the total number of genes in the genome for each biological process was also calculated (background).

Arabidopsis Col-0 wild-type and *atg5* mutants displayed a large difference in gene expression profiles prior to heat treatment (Fig 2B). To understand the effects of autophagy on plant gene expression under normal temperature and how these effects affected subsequent heat-regulated gene expression, we first performed GO enrichment analysis to assign biological processes to the identified DEGs between Col-0 and *atg5* at 0 hour of heat treatment. As shown in Fig 3A, among up-regulated DEGs in the *atg5* mutant plants, those involved in cell division, organelle fission, nuclear division, mitotic cell cycle and DNA replication were significantly enriched. Intriguingly, a substantial number of up-regulated DEGs are associated closely with biological processes directly or indirectly related to DNA and RNA synthesis, metabolism and modification. These biological processes include DNA metabolic process, RNA processing, cellular response to DNA damage stimuli, DNA repair, chromatin medication and DNA replication. On the other hand, among the DEGs down-regulated in the *atg5* mutant, those involved in responses to endogenous or environmental stimuli were substantially enriched (Fig 3B). These environmental stimuli include water deprivation, light, radiation and temperature (Fig 3B). Therefore, the GO analysis of the DEGs revealed that under normal growth temperature, deficiency of autophagy caused increased expression of genes involved in regulation of cell division and growth but reduced expression of genes involved in responses to endogenous and, particularly, environmental stimuli.

**Fig 4:**
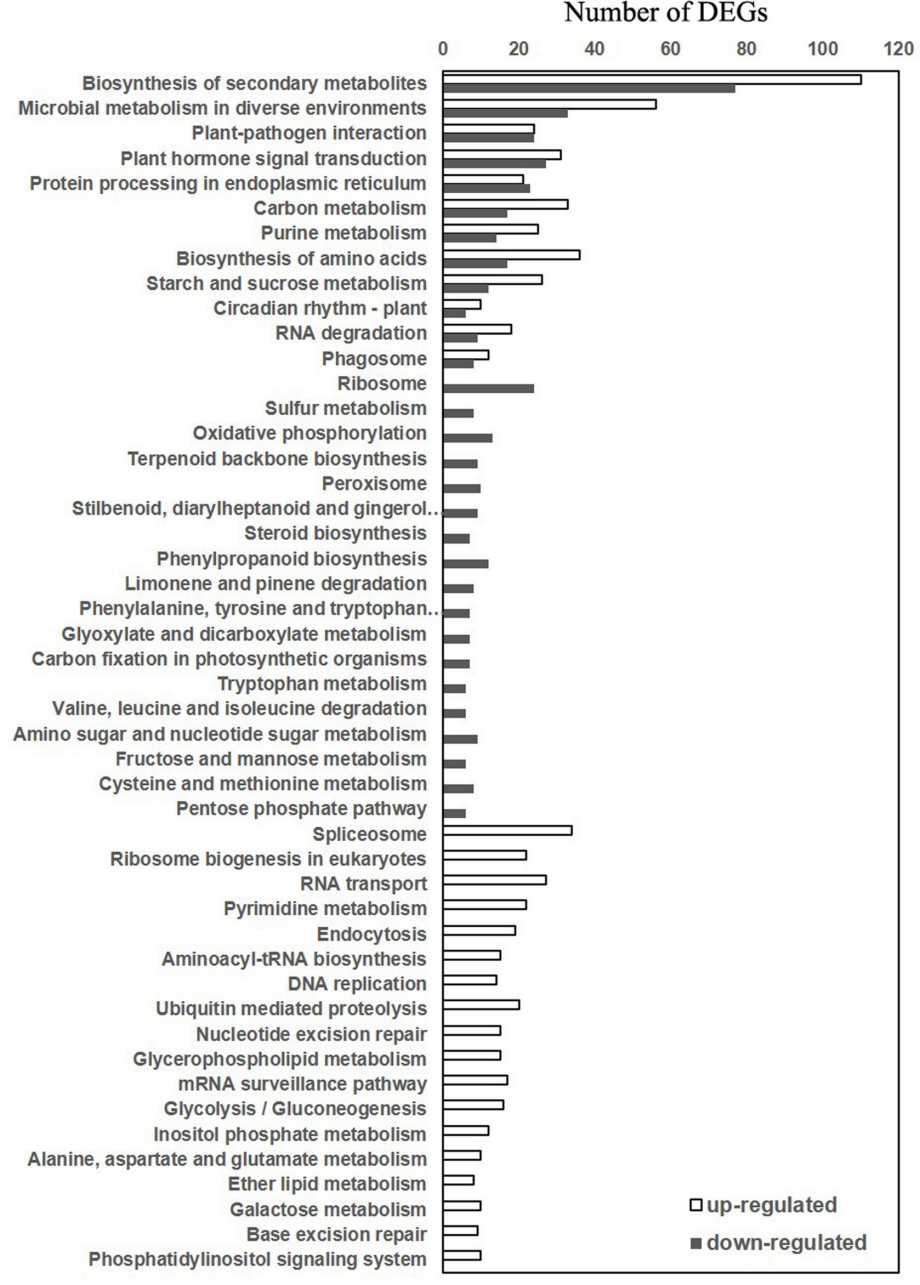
KEGG pathway analysis of DEGs between Col-0 and *atg5* mutant at 0 hour of heat treatment. Both the numbers of both up- and down-regulated DEGs in the *atg5* mutant are shown.

We have also performed KEGG pathway enrichment analysis of the DEGs between wild-type and *atg5* mutant prior to heat treatment (S3 Fig) and DEGs between 0 and 3 (S4 Fig), 0 and 6 (S5 Fig) and 0 and 9 (S6 Fig) hours of heat treatment in wild-type and *atg5* mutant. those DEGs with the highest statistical significance in KEGG enrichment were subjected to further analysis. The analysis revealed that these DEGs could be grouped into three categories of pathways. The first category contained both up-and down-regulated DEGs with roles in biosynthesis of secondary metabolites, plant-pathogen interaction, hormone signaling, ER protein processing and metabolism of carbohydrates and amino acids (Fig 4). The second category contained only down-regulated DEGs involved in biosynthesis and metabolism of a variety of primary and secondary metabolites such as terpenoids, steroids, phenylpropanoids, glyoxylates and dicarboxylates (Fig 4). The second category also include down-regulated DEGs involved in the degradation and metabolism of a substantial number of amino acids including phenylalanine, tyrosine, tryptophan, valine, leucine, isoleucine, cysteine and methionine (Fig 4). The third category contained only up-regulated DEGs with roles in nucleotide biosynthesis, transport, repair and metabolism that are associated with cell division and growth regulation (Fig 4). A number of primary metabolic pathways including glycolysis, gluconeogenesis, metabolism of alanine, aspartate and glutamate also belonged to this category (Fig 4). Taken together, the KEGG analysis supports the finding from the GO enrichment analysis that those genes involved in cell division, cell cycle regulation, DNA repair and RNA processing were up-regulated in the *atg5* mutant under normal growth conditions (Fig 4). While the GO analysis revealed down-regulation of genes involved in stress responses in the *atg5* mutant plants (Fig 3), the KEGG analysis further indicated down-regulation of genes involved in metabolism of a variety of amino acids in the *atg5* mutants under normal growth conditions (Fig 4).

### GO analysis of DEGs between Col-0 and *atg5* mutant in response to heat stress

**Fig 5:**
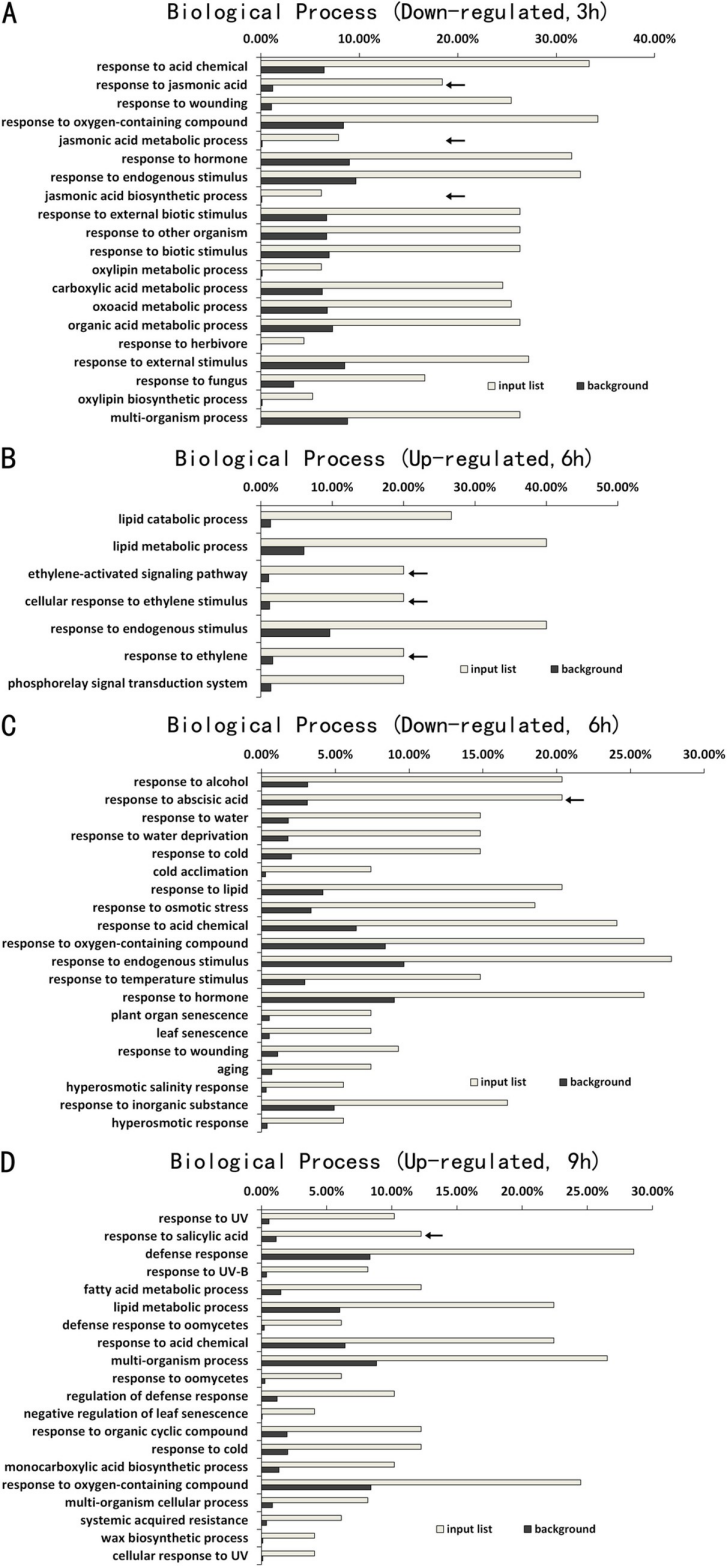
GO enrichment analysis of DEGs between Col-0 and *atg5* mutant at 3, 6 and 9 hours of heat treatment. The enrichment analysis was performed using the GO term that describes biological process for down-regulated DEGs at 3 hours (**A**), up-regulated DEGs (**B**) and down-regulated DEGs at 6 hours (**C**), and up-regulated DEGs at 9 hours (**D**) of heat treatment. No significant enrichment was found for up-regulated DEGs at 3 hours and down-regulated DEGs at 9 hours of heat treatment. Calculation of input list and background DEG numbers for each biological process was performed as in Fig 3.

As described earlier, the numbers of DEGs between Col-0 and *atg5* mutant at 3, 6 and 9 hours of heat treatment were much smaller than those at 0 hour of heat treatment (Fig 2B). To gain insights into the DEGs, we performed GO enrichment analysis to determine the specific biological processes in which these genes are involved. There were only 28 up-regulated DEGs in the *atg5* mutant at 3 hours of heat treatment and GO analysis revealed no enrichment of any particular biological process for these DEGs. On the other hand, of the 135 DEGs down-regulated in the *atg5* mutant at 3 hours of heat treatment, a majority of them were assigned to the biological processes of responses to stress-related stimuli including jasmonic acid (JA), wounding, pathogens, herbivore and other organisms (Fig 5A). At 6 hours of heat stress, there were 22 DEGs up-regulated in the *atg5* mutant with an enrichment in the biological processes in lipid metabolism and ethylene signaling (Fig 5B). On the other hand, of the 86 DEGs down-regulated in the *atg5* mutant at 6 hours of heat stress, a majority of them were again assigned to the biological processes of responses to biotic and abiotic stresses (Fig 5C). By contrast, a majority of DEGs up-regulated in the *atg5* mutant at 9 hours of heat stress were involved in plant defense and stress responses (Fig 5D). Interestingly, there were only 22 DEGs down-regulated in the *atg5* mutant at 9 hours of heat treatment and GO analysis revealed no enrichment of any particular biological process for these DEGs. Taken together, the GO analysis of heat-regulated DEGs indicated that autophagy played a positive role in the expression of defense- and stress-related genes at 3 and 6 hours of heat stress but reduced their expression at the late stage of heat stress.

### Modulation of autophagy-regulated gene expression by heat stress

**Fig 6:**
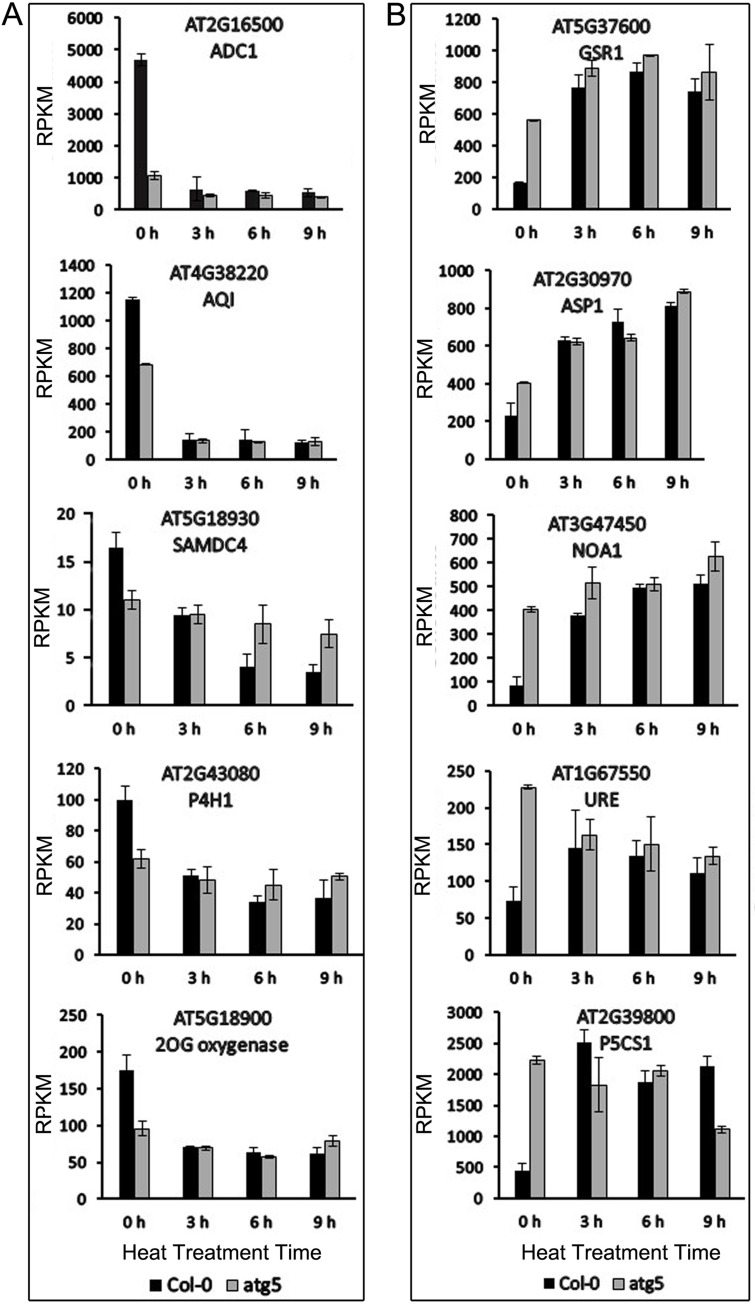
Expression analysis of genes involved in amino acid arginine and proline metabolism. Five representative genes each from two groups, one down-regulated (A) and the other up-regulated (B) in the *atg5* mutant at 0 hour of heat treatment, were analyzed for change of expression after heat stress. RPKM expression values for the genes at indicated hours of heat treatment are shown.

Both PCA and DEG analysis revealed that the gene expression profiles between Col-0 and *atg5* mutant were more different at 0 hour than at 3, 6 or 9 hours of heat stress. Thus, heat stress appeared to mask, to a great extent, the effects of autophagy on plant gene expression. To understand how heat stress modulated autophagy-regulated gene expression, we analyzed the 40 identified DEGs involved in amino acid arginine and proline metabolism after heat treatment. Among the 40 DEGs, 11 were down-regulated and 18 up-regulated in the *atg5* mutant at 0 hour of heat treatment. We chose representative genes from these two groups and analyzed the changes of their transcript levels after heat treatment. The first group of 5 genes were selected from those that were down-regulated in the *atg5* mutant at 0 hour of heat treatment (Fig 6A). Upon 3 hours of heat stress, their transcript levels in Col-0 were all substantially reduced and remained low after 6 and 9 hours of heat stress (Fig 6A). On the other hand, the transcript levels for these genes were reduced only modestly in the *atg5* mutants after heat stress (Fig 6A). As a result, the transcript levels for the genes became similar between Col-0 and *atg5* mutant after heat treatment (Fig 6A). The second group of 5 genes were up-regulated in the *atg5* mutant at 0 hour of heat treatment. At 3 hours of heat stress, their transcript levels were all substantially induced in Col-0 and remained high at 6 and 9 hours of heat stress (Fig 6B). In the *atg5* mutant, on the other hand, the transcript levels for the genes were induced only modestly or not induced at all after heat stress (Fig 6B). Because the stronger heat induction offset the lower expression levels for the genes, their transcript levels in Col-0 again became similar to those in *atg5* mutant after heat treatment (Fig 6).

**Fig 7:**
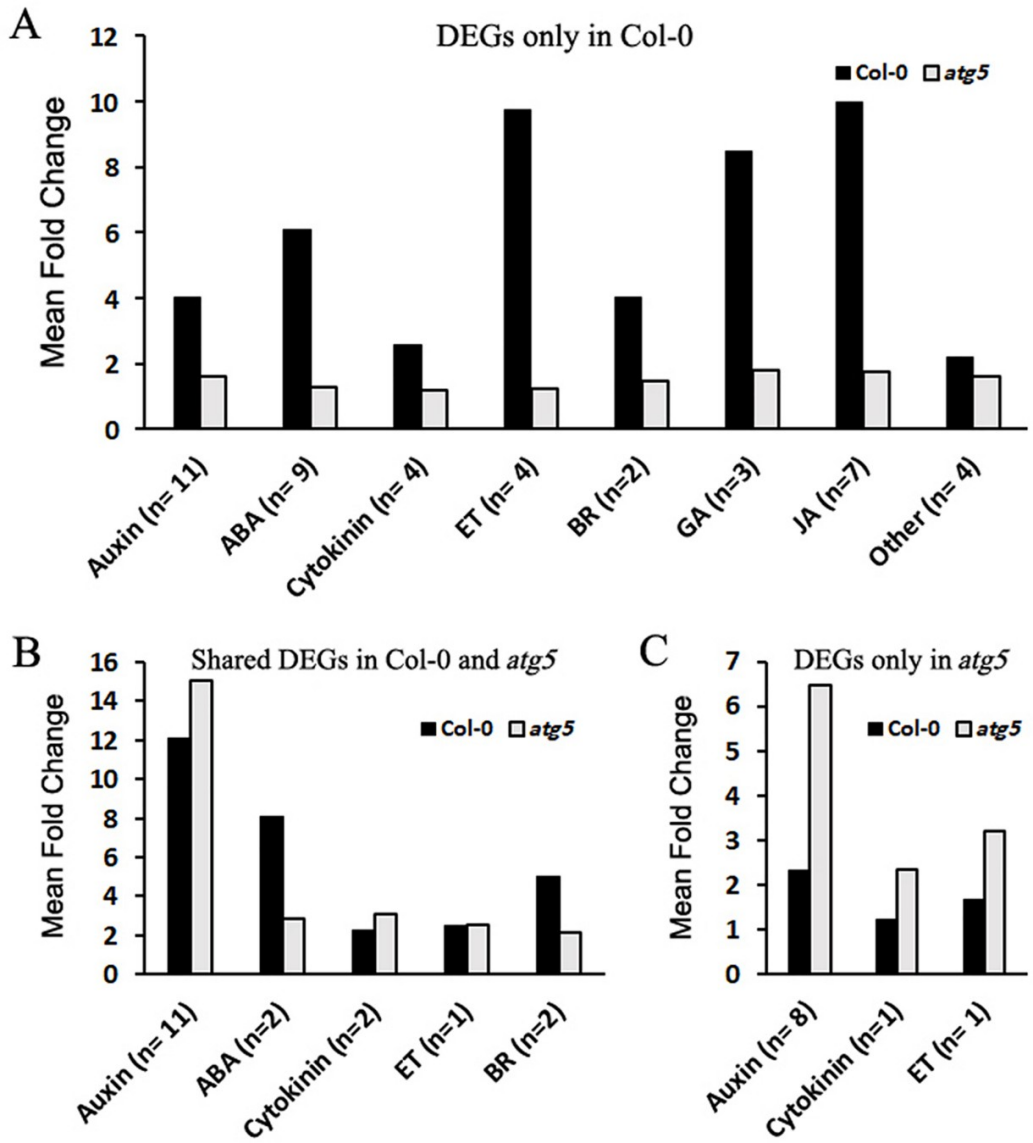
Analysis of heat-regulated DEGs associated with phytohormone signaling. A total of 72 heat-regulated DEGs between 0 and 3 hours of heat stress were assigned to signaling pathways of specific plant hormones from KEGG pathway analysis. Of these heat-regulated DEGs, some were differentially expressed in response to heat stress only in Col-0 (**A**), in both Col-0 and *atg5* mutant (**B**) or only in *atg5* mutant (**C**). The numbers of DEGs for each specific plant hormone (n) and their average fold change of expression levels after 3 hours of heat stress are shown.

To further analyze the effects of heat stress on autophagy-regulated genes, we compared Col-0 and *atg5* mutant for heat-regulated DEGs associated with phytohormone signaling. KEGG pathway analysis identified a total of 72 DEGs that were assigned to signaling pathways of specific plant hormones. Among the 72 DEGs, 44 displayed an average of 5.8-fold change in Col-0 but showed little change in expression in the *atg5* mutant after 3 hours of heat stress (Fig 7A and S2 Table). In comparison, there were 18 hormone signaling-related DEGs with similar levels of change in expression in both Col-0 and *atg5* mutant after heat stress (Fig 7B and S3 Table). Only 10 plant hormone signaling-related DEGs were identified in the *atg5* mutant but not in Col-0 (Fig 7C and S4 Table). These results further indicated that in the autophagy-deficient *atg5* mutant, the magnitude of heat-regulated gene expression was substantially reduced.

### Modulation of JA-regulated genes by both autophagy and heat stress

**Fig 8:**
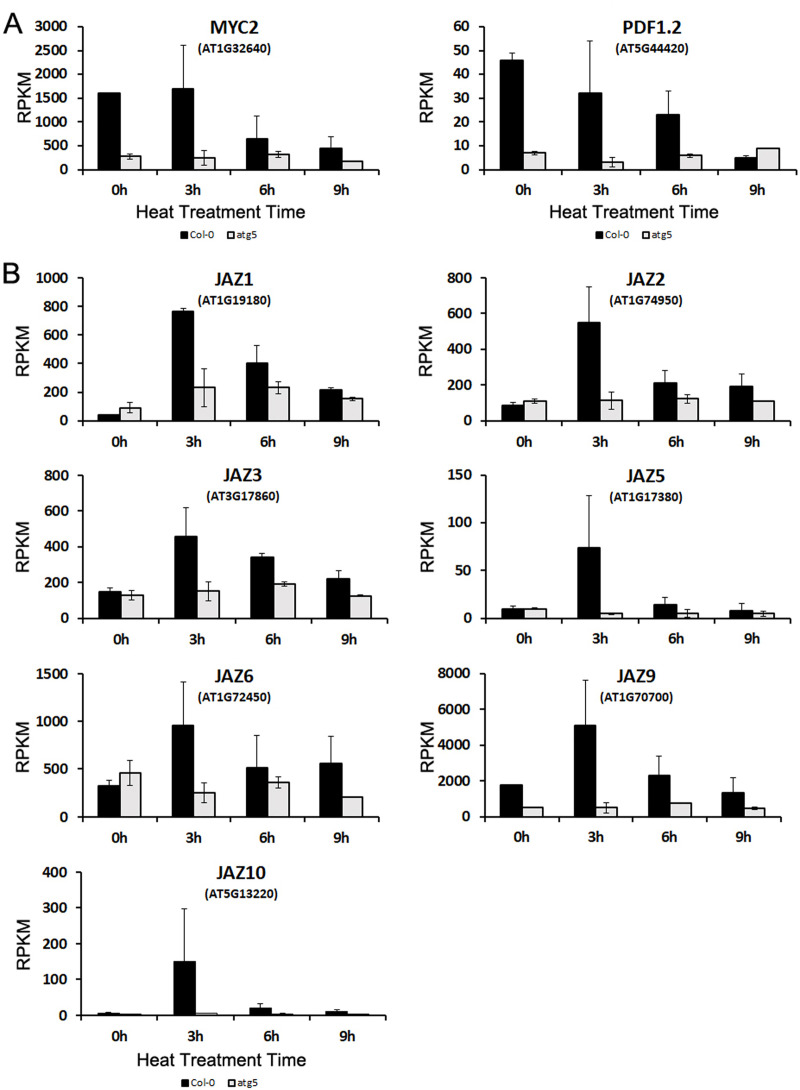
Expression analysis of genes involved in JA signaling. RPKM expression values for the *MYC2* and *PDF1*.*2* (A) and JAZ (B) genes at indicated hours of heat treatment are shown.

Arabidopsis MYC2 is a transcription factor that regulates diverse JA-dependent functions [58]. Analysis of transcriptome profiles revealed that the transcript levels of *MYC2* in Col-0 plants were about 5 times higher than those in the *atg5* mutant before heat treatment (Fig 8A), suggesting a role of autophagy in modulating JA signaling. Indeed, the transcript levels of *PDF1*.*2*, a JA signaling marker, were also 5–7 times higher in the wild-type plants than in the *atg5* mutant at 0 hour of heat treatment (Fig 8A). MYC2 is a negative regulator of tryptophan metabolism and biosynthesis of tryptophan-derived secondary metabolites but is a positive regulator of flavonoid biosynthesis [58]. The reduced expression of the transcription factor in the *atg5* mutant could account for altered expression of genes involved in these metabolic pathways as revealed from the comparative transcriptome profiling of the wild-type and *atg5* mutant under normal growth conditions (Figs 3 and 4). The transcript levels of *MYC2* remained high after the first 3 hours of heat stress but declined with extended time at the high temperature in the wild type (Fig 8A). Heat stress also led to decline in the transcript levels of *PDF1*.*2* in the wild-type plants (Fig 8A). The transcript levels of both *MYC2* and *PDF1*.*2* remained consistently low throughout the entire 9 hours of heat stress (Fig 8A).

JASMONATE ZIM-DOMAIN (JAZ) proteins are negative regulators of JA signaling by repressing JA-responsive genes through direct binding and suppression of the activity of MYC2 and its closely related paralogs [59]. *JAZ* genes are known to be induced by JA, most likely as a negative feedback mechanism for regulation of JA signaling [60]. Despite the large difference in transcript levels of both *MYC2* and *PDF1*.*2* between wild-type and *atg5* mutant plants prior to heat stress, the transcript levels of JAZ genes were very similar between the two genotypes (Fig 8B). During the first 3 hours of heat stress, the transcript levels of the *JAZ* genes were all strongly induced in the wild-type plants (Fig 8B). The strong heat induction of the *JAZ* genes was correlated with the gradual repression of JA-repressive *PDF1*.*2* gene in heat-treated wild-type plants (Fig 8A). Furthermore, heat-induced expression of *JAZ* genes was transient as their transcripts declined rapidly with extended period of heat stress (Fig 8B). As with *MYC2* and *PDF1*.*2*, expression of these *JAZ* genes in the *atg5* mutant was generally low and relatively stable throughout the entire 9 hours of heat stress (Fig 8B). Thus, both autophagy and heat stress modulated the expression of regulatory genes of JA signaling and JA-responsive target genes.

### Effect of autophagy on nuclear heat-responsive gene expression

**Fig 9:**
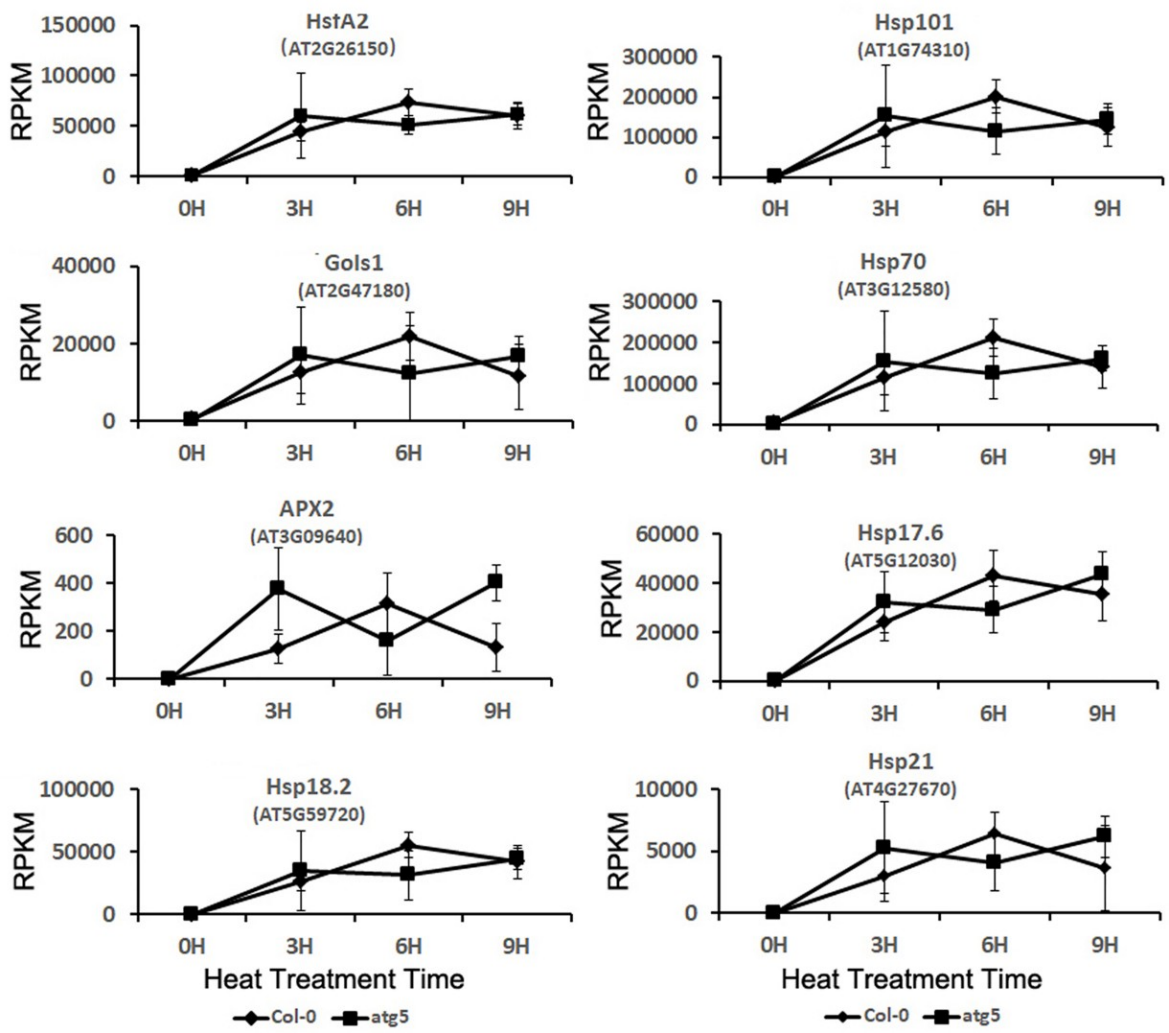
Expression analysis of heat shock transcription factor gene *HsfA2* and its target genes. RPKM expression values for the genes at indicated hours of heat treatment are shown.

HSPs, including Hsp101, Hsp70, and small HSPs act as molecular chaperones in protein quality control by promoting the folding and refolding of nonnative proteins [2–6]. The genes encoding HSPs are rapidly induced by heat in plants. Heat shock transcription factors (HSFs) mediate the expression of HSPs and other heat-induced genes [61]. In Arabidopsis, HsfA2 plays a key role in the induction of plant heat stress responses [61]. Comparison of transcriptome profiles revealed similar basal transcript levels of Arabidopsis *HsfA2* in Arabidopsis wild-type and *atg5* mutant (Fig 9). The transcript levels of *HsfA2* in both the wild type and *atg5* mutants increased rapidly during the first three hours at 45°C and remained highly induced during the entire 9 hours at the high temperature (Fig 9). Even though the *HsfA2* transcript levels were slightly higher in the *atg5* mutant than those in wild type after the first 3 hour of heat stress, they also declined slightly earlier in the autophagy-deficient mutant (Fig 9). Therefore, the induction of the heat shock transcription factor gene was in general very similar in the wild-type and atg5 mutant (Fig 9). We also analyzed a subset of HsfA2 target genes for their expression before and after heat treatment. These target genes encode APX2, GolS1, and several HSPs (Hsp17.6, Hsp18.2, Hsp21, Hsp70, and Hsp101). We again observed that these HsfA2 target genes were strongly induced in the wild type and *atg5* mutant (Fig 9). As with *HsfA2*, even though there was small difference in the kinetics of induction for the target genes, their overall patterns of induction after heat stress were generally very similar in the wild-type and *atg5* mutant (Fig 9). Thus, autophagy deficiency in the *atg5* mutant had little effect on both basal and induced expression of *HsfA2* and its target genes. The rapid and strong induction of *HsfA2* and the target genes of HSFA2 by heat treatment in both wild-type and *atg5* mutant plants, which are highly consistent with the expression patterns of these genes from other reported studies and, therefore, is a strong validation of the transcriptomic data of the study.

### Effect of autophagy on heat-regulated expression of transcription factor genes

**Fig 10:**
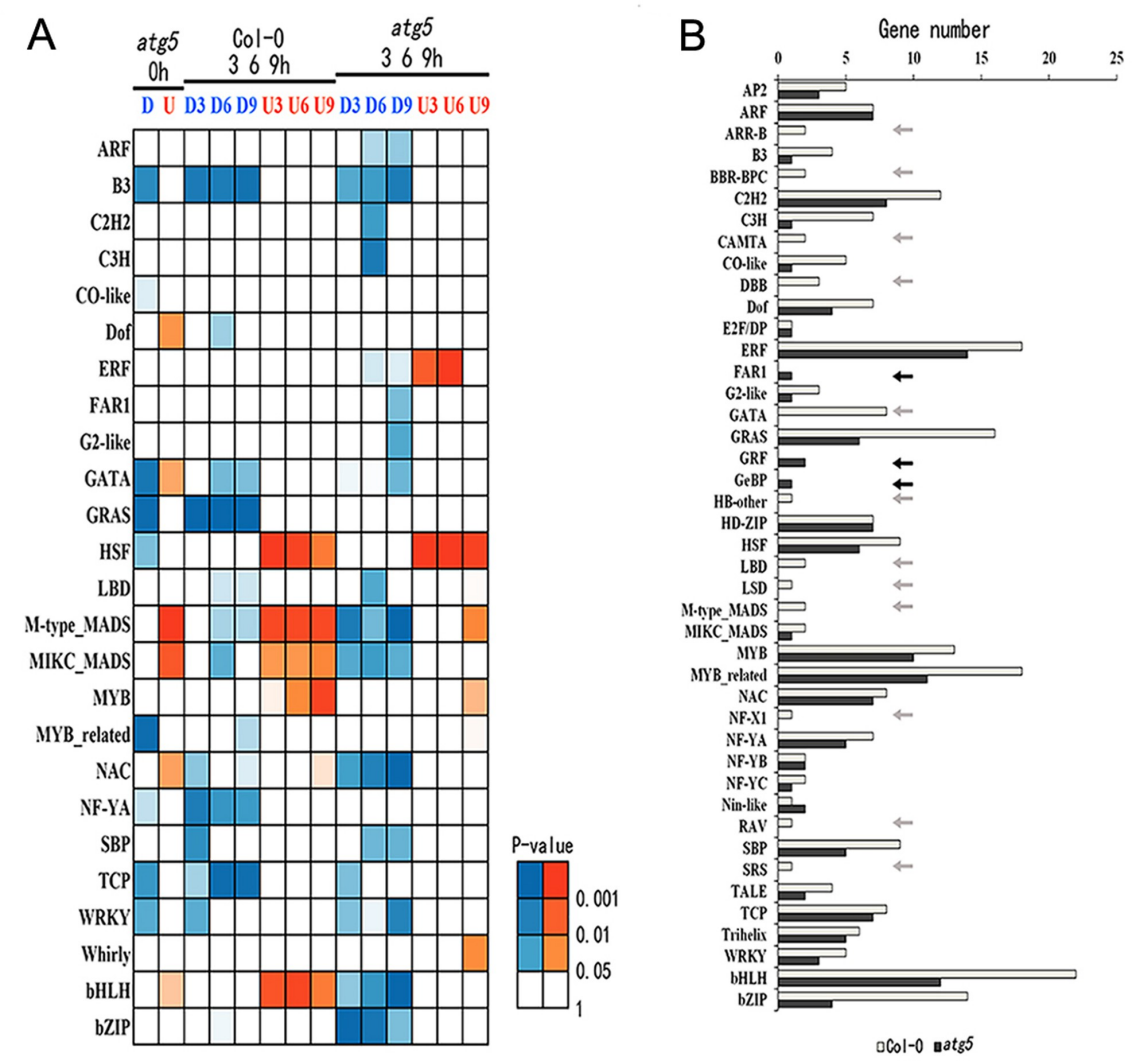
Differential expressed genes encoding transcription factors. A. Up- and down-regulated DEGs of TF genes were identified through comparison of expression values between Col-0 and *atg5* at 0 hour of heat treatment or between heat treated and untreated plants in the Col-0 or *atg5*. Differences of the expression value were statistically analyzed and the different P-values are indicated in different colors. B. Number of heat-regulated core TF DEGs from Col-0 and *atg5*.

Sequence-specific transcription factors are the most important regulators of gene expression. To gain insights into the regulatory network of autophagy- and heat-regulated gene expression, we also analyzed the transcriptome data for the differential expression of genes encoding members of major plant transcription factor families. From the analysis, we identified members from a number of transcription factor families including B3, GRAS, MYB-related, TCP and WRKY were downregulated while members from Def, MADS and NAC were upregulated in the *atg5* mutant at 0 hour of heat stress (Fig 10A). During heat treatment, members of several transcription factor families were differentially regulated between Col-0 and *atg5* mutant. These differentially expressed transcription factors belong to families including ERF, MADS, NAC, bNLH and bZIP (Fig 10A). Notably, some transcription factor families such as HSF showed relatively subtle difference in their expression between the Col-0 and *atg5* mutant during the period of heat stress (Fig 10A). We also analyzed the numbers of transcription factor genes whose expression was altered by heat stress and again found a major effect of autophagy on heat-regulated expression of genes encoding members of several transcription factor families (Fig 10B).

## Discussion

Autophagy plays a critical role in plant tolerance to heat stress, which causes many harmful effects on cellular components and structures including misfolding, denaturing and aggregation of proteins. Therefore, studies on the role of autophagy in plant heat tolerance have been mostly focused on autophagy as a protein degradation pathway that targets heat stress-related proteins such as protein aggregates and chaperones for degradation during heat stress [9–11]. However, as a catabolic pathway, autophagy targets not only heat-related proteins but also other excess, aggregated, or damaged proteins and organelles to the lysosomes or vacuole to impact metabolism, signaling, gene expression and other processes, which can also influence plant heat tolerance. To examine the broad function of autophagy in plant heat stress responses, we analyzed the changes of transcriptomes of Arabidopsis wild-type and autophagy-deficient *atg5* mutant in response to heat stress. From the comparative transcriptome profiling we have made several findings with important implications in the understanding of the molecular and functional relationship between autophagy, plant heat stress responses and heat tolerance. First, we found that autophagy deficiency caused altered expression of thousands of Arabidopsis genes even prior to heat treatment (Fig 2). Importantly, heat treatment caused more robust change in transcript profiles in the wild-type plants than in the *atg5* mutant (Fig 2) and this differential effect appeared to be associated with the fact that the expression of a large number of heat-regulated genes was already altered prior to heat stress and became less responsive to heat stress in the *atg5* mutant (Figs 6 & 7). Thus, deficiency of autophagy and heat stress appeared to influence a large set of plant genes in a similar manner, indicating a physiological stress in the autophagy-deficient mutant plants even under normal growth conditions. The reduced responsiveness of a large set of heat-regulated plant genes to heat stress in the autophagy-deficient mutant plants likely reflects a compensatory mechanism for maintaining cellular homeostasis. However, the chronical state of altered expression of a large set of heat-regulated genes even under normal growth conditions in the autophagy-deficient mutants could negatively affect plant fitness and the reduced responses of these genes to high temperature could also hamper plant response and adaptation to heat stress.

A majority of genes whose expression was affected by autophagy prior to heat stress encode proteins involved in metabolism, stress responses and signaling and can shape or condition plants for response to heat stress (Figs 3 & 4). Among the metabolic genes affected by autophagy are those involved in the biosynthesis and metabolism of amino acids and secondary metabolites (Figs 3 and 4). As constituents of proteins, biosynthesis and metabolism of amino acids are closely linked with protein biosynthesis, a fundamental biological process that provides the driving force not only for growth and development but also for responses to heat stress including the production of heat shock proteins. In addition, sulfur-containing amino acids methionine and cysteines are precursors of glutathione, a key water-soluble antioxidant and plays a central part in scavenging of reactive oxygen species (ROS), which is often elevated under stress conditions including heat stress [62]. Likewise, some plant secondary metabolites function as osmolytes, antioxidants and growth precursors to help plants recover from heat stress, while other metabolites help protect membranes from harmful effects from heat stress [63]. In addition, a substantial number of up-regulated DEGs in the *atg5* mutant are associated closely with biological processes in the nucleus including DNA replication, repair and metabolism, and chromatin modification (Figs 3 and 4). The physiological relevance of these molecular processes in plant heat stress responses is unclear but some of them such as chromatin modification could alter gene expression when plants are exposed to heat stress. Up-regulation of genes involved in DNA repair and responses to DNA damage stimuli could also indicate a role of autophagy in maintaining genome stability, which has been well established in other eukaryotic organisms [64, 65].

Many genes involved in metabolism and signaling were highly responsive to heat treatment in wild-type plants but less responsive in the *atg5* mutant, particularly during the early stage of heat stress (3 and 6 hours of heat treatment) (Figs 1 & 2). Many of these heat-responsive genes were already altered in expression in the *atg5* mutant when compared to that in the wild-type plants prior to heat stress (Fig 6). Therefore, these genes were not as responsive to heat stress during the early hours of heat stress in the *atg5* mutant as in the wild-type plants, most likely due to a feedback mechanism. However, with extended hours of heat stress (e.g. 9 hours of heat treatment), these effects of heat effects may overtake or even overwhelm the effect of autophagy and, therefore, the DEG numbers between WT and *atg5* mutant became more similar.

Even though the numbers of DEGs between Col-0 and *atg5* mutant reduced drastically upon heat stress (Fig 2B), the nature of these genes and the way of their regulation provided important clues as to how autophagy modulate plant gene expression during heat stress. A majority of the DEGs identified between Col-0 and *atg5* mutant after 3, 6 and 9 hours of heat stress are involved in responses to wounding, pathogens, herbivores, water deprivation, temperature, osmotic conditions and stress-related plant hormones (Fig 6). This is in direct contrast to the DEGs identified between Col-0 and *atg5* mutant prior to heat treatment, of which a large percentage are involved in metabolism (Figs 3 & 4). The differential expression of these defense-related and stress-responsive genes in heat-treated *atg5* mutants could be indictive of an active role of autophagy in modulation of heat-regulated gene expression to promote plant heat tolerance. Alternatively, altered expression of the defense and stress genes in the *atg5* mutant under heat stress might be due to the consequence of compromised heat tolerance of the *atg5* mutant under heat stress. Significantly, the defense- and stress-related DEGs identified between Col-0 and a*tg5* mutant after 3 and 6 hours of heat treatment were mostly down-regulated in the *atg5* mutants (Fig 6). On the other hand, the defense- and stress-related DEGs identified between Col-0 and *atg5* mutant after 9 hours of heat treatment were mostly upregulated in the *atg5* mutant (Fig 6), most likely in response to more severe stressed state of the *atg5* mutant under prolonged heat stress. Thus, during the relatively early stages of heat stress (e.g. at 3 and 6 hours of heat stress), autophagy plays a positive role in the expression of defense- and stress-related genes, which could provide a new mechanism by which autophagy promotes plant heat tolerance.

Even though autophagy modulates expression of a spectrum of stress-responsive genes, it had little effect on the rapid induction of the gene encoding the key heat shock transcription factor HsfA2 (Fig 9). Likewise, the rapid and strong induction of HsfA2 target genes was also normal in the *atg5* mutant after heat stress (Fig 9). Many of the HsfA2 target genes encode HSPs that function as protein chaperones and co-chaperones with important roles in protein folding, refolding and quality control. As two critical processes in the cellular protein quality control networks, heat shock responses and autophagy cooperate in maintaining protein homeostasis [66]. Some of HSPs are also subjected to degradation by autophagy for re-setting cellular biochemistry once the heat stress ends for recovery of plant growth [42]. Therefore, despite normal induction of heat shock genes in the autophagy-deficient mutant plants, autophagy is likely to have an important role as a close partner of heat shock responses in promoting protein homeostasis of plant cells under heat stress.

In summary, we have performed transcriptome profiling of Arabidopsis wild-type and autophagy-deficient *atg5* mutant prior to and after heat stress to assess the broad role of autophagy in plant heat stress response. We have discovered major transcriptomic alterations between wild-type and *atg5* mutant under normal growth conditions that affect expression of a large number of genes involved in metabolism, hormone signaling, stress responses, cell cycle, nucleotide processing and DNA repair. The altered expression of the genes involved in metabolism and other biological processes could shape, prime or condition plants for response to encountered heat stress conditions. In addition, autophagy had little effect on heat-induced expression of heat shock genes but played a positive role in the expression of a large number of other defense- and stress-related genes during the early stage of heat stress responses. These findings provide important new insights into the broad role of autophagy and enhance our understanding of the complex networks of plant heat stress responses.

## Supporting information

S1 Fig Mapped reads of RNA-seq data. (TIF)

S2 Fig Distribution of total genes expression. (TIF)

S3 Fig KEGG pathway enrichment analysis of DEGs between Col-0 wild-type and *atg5* mutant at 0 hour of heat treatment. (TIF)

S4 Fig KEGG pathway enrichment analysis of DEGs between 0 and 3 hours of heat treatment in Col-0 wild-type and *atg5* mutant. (TIF)

S5 Fig KEGG pathway enrichment analysis of DEGs between 0 and 6 hours of heat treatment in Col-0 wild-type and *atg5* mutant. (TIF)

S6 Fig KEGG pathway enrichment analysis of DEGs between 0 and 9 hours of heat treatment in Col-0 wild-type and *atg5* mutant. (TIF)

S1 Table Pearson’s Correlation Coefficient (r^2^) analysis of the sample replicates. (TIF)

S2 Table Heat-regulated DEGs associated with phytohormone signaling only in Col-0. (TIF)

S3 Table Heat-regulated DEGs associated with phytohormone signaling in both Col-0 and *atg5* mutant. (TIF)

S4 Table Heat-regulated DEGs associated with phytohormone signaling only in *atg5* mutant. (TIF)
