# Supplementary figures and images for: Transcriptome analysis of the role of autophagy in plant response to heat stress
Source: PLoS One. 2021 Feb 26;16(2):e0247783. doi: 10.1371/journal.pone.0247783 (PMC7909648; doi:10.1371/journal.pone.0247783)

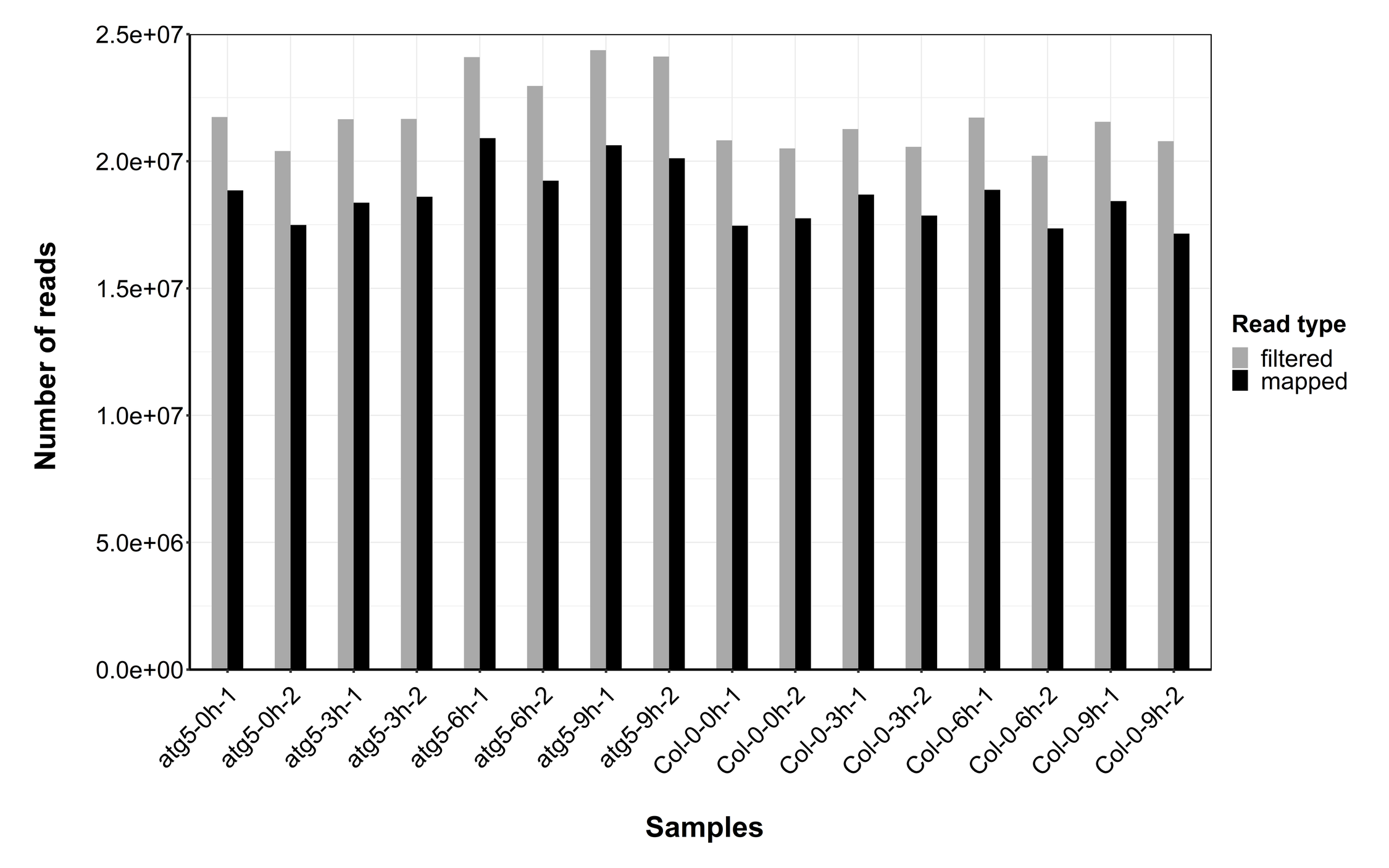

Supplement: S1 Fig — (TIF) [file pone.0247783.s001.tif]

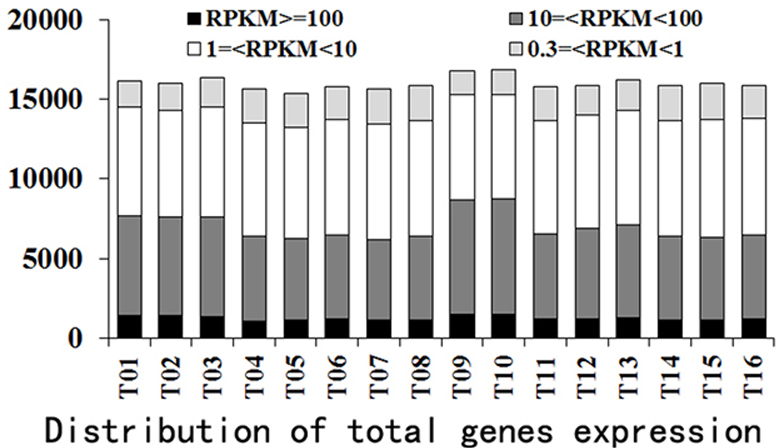

Supplement: S2 Fig — (TIF) [file pone.0247783.s002.tif]

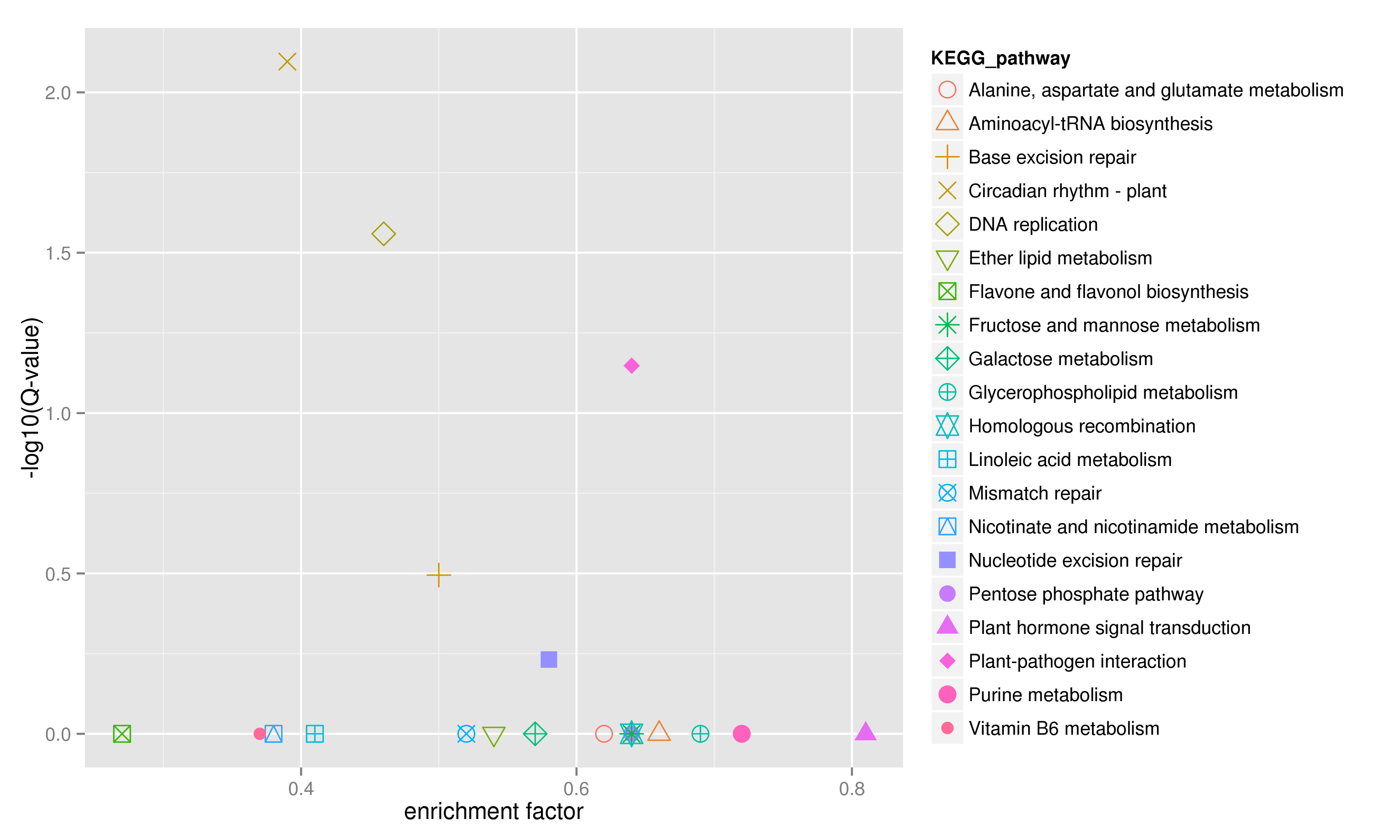

Supplement: S3 Fig — (TIF) [file pone.0247783.s003.tif]

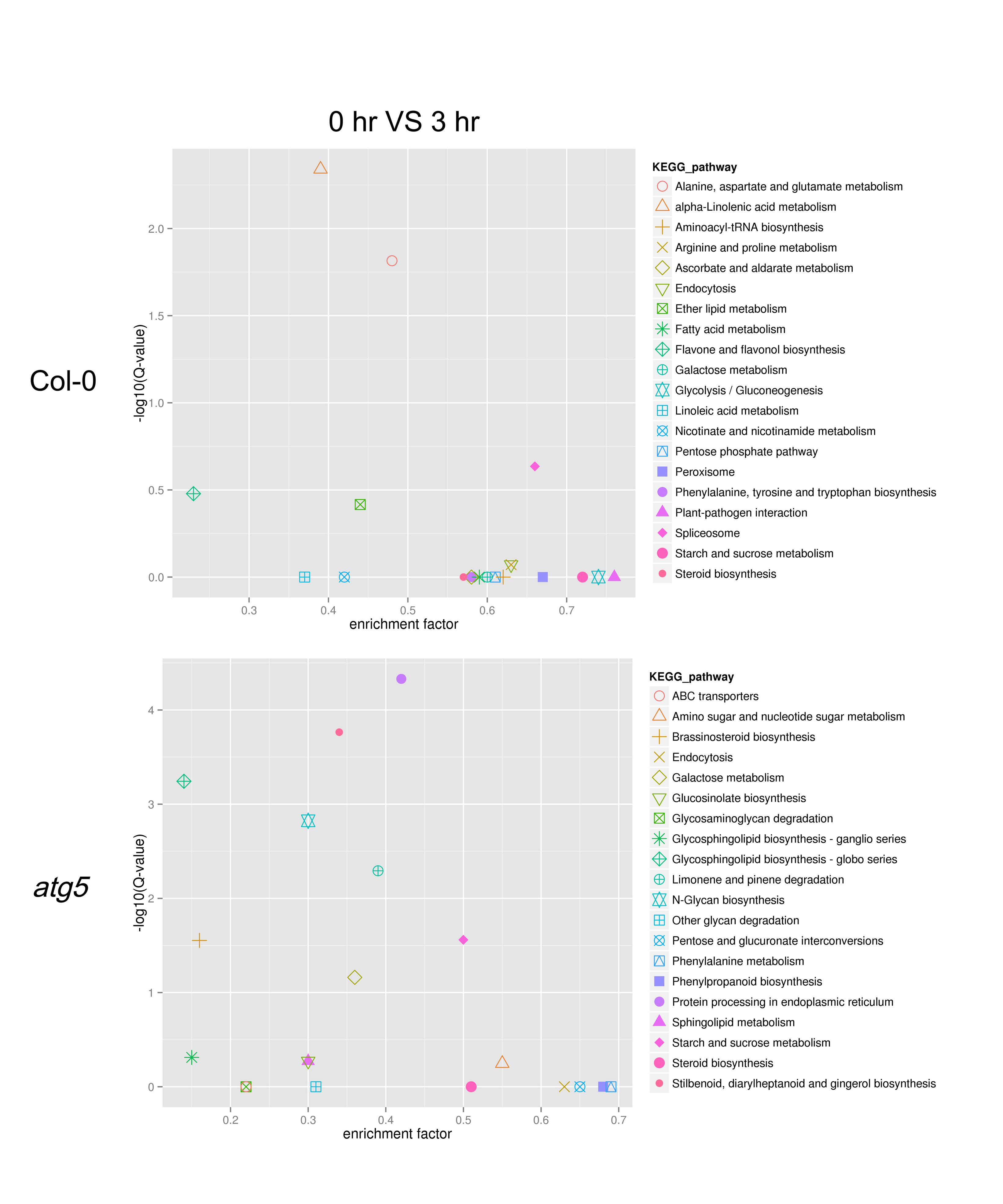

Supplement: S4 Fig — (TIF) [file pone.0247783.s004.tif]

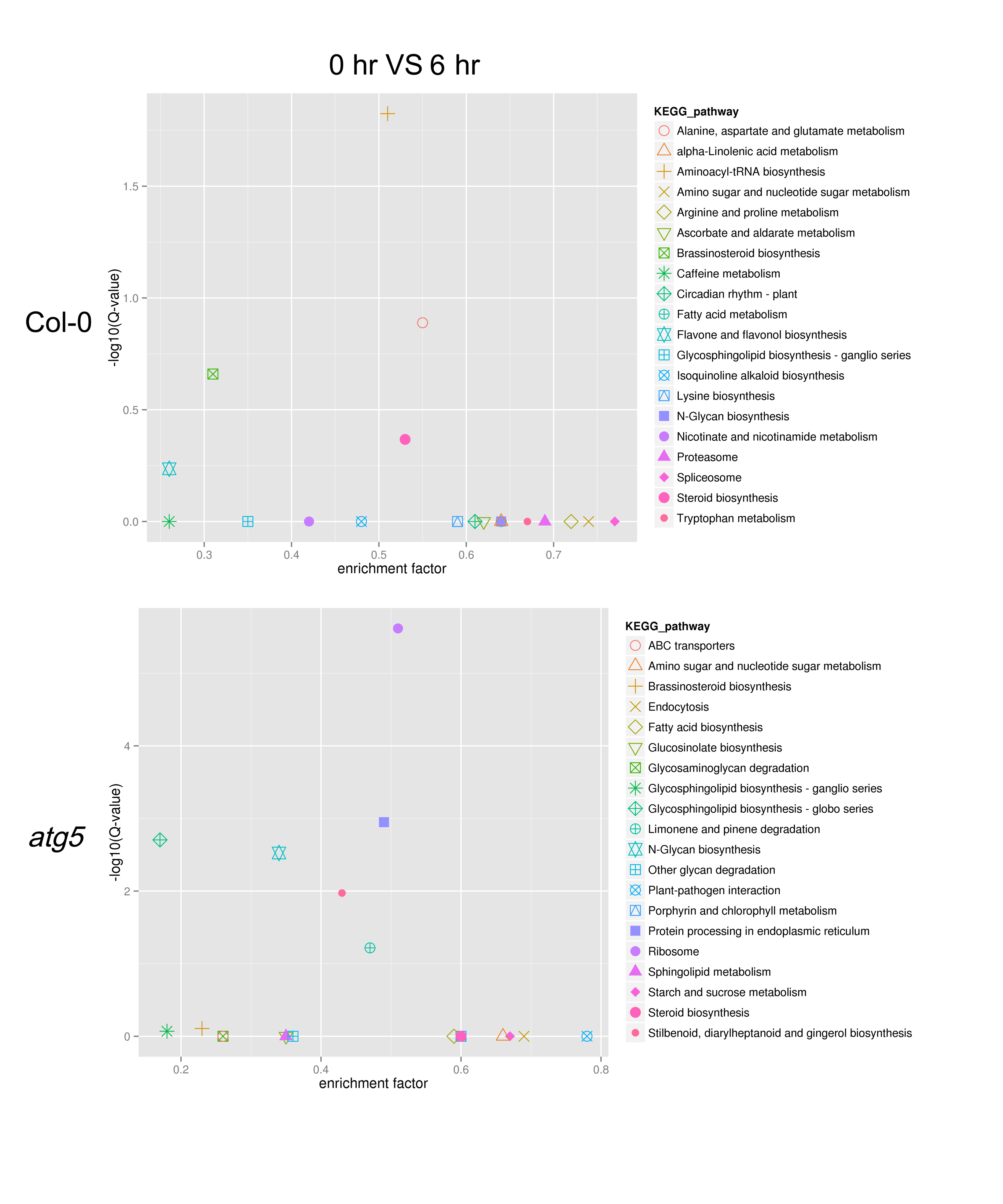

Supplement: S5 Fig — (TIF) [file pone.0247783.s005.tif]

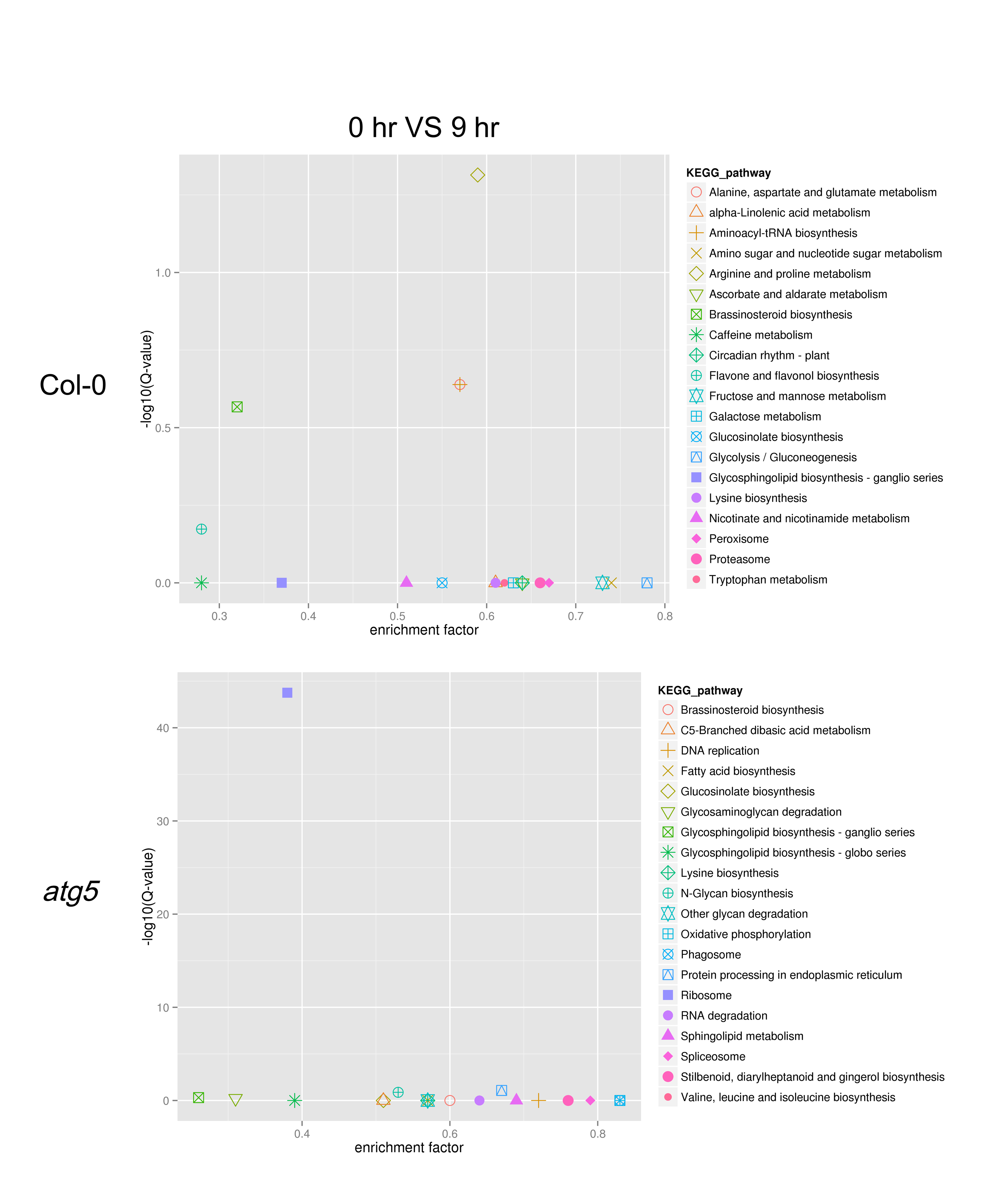

Supplement: S6 Fig — (TIF) [file pone.0247783.s006.tif]

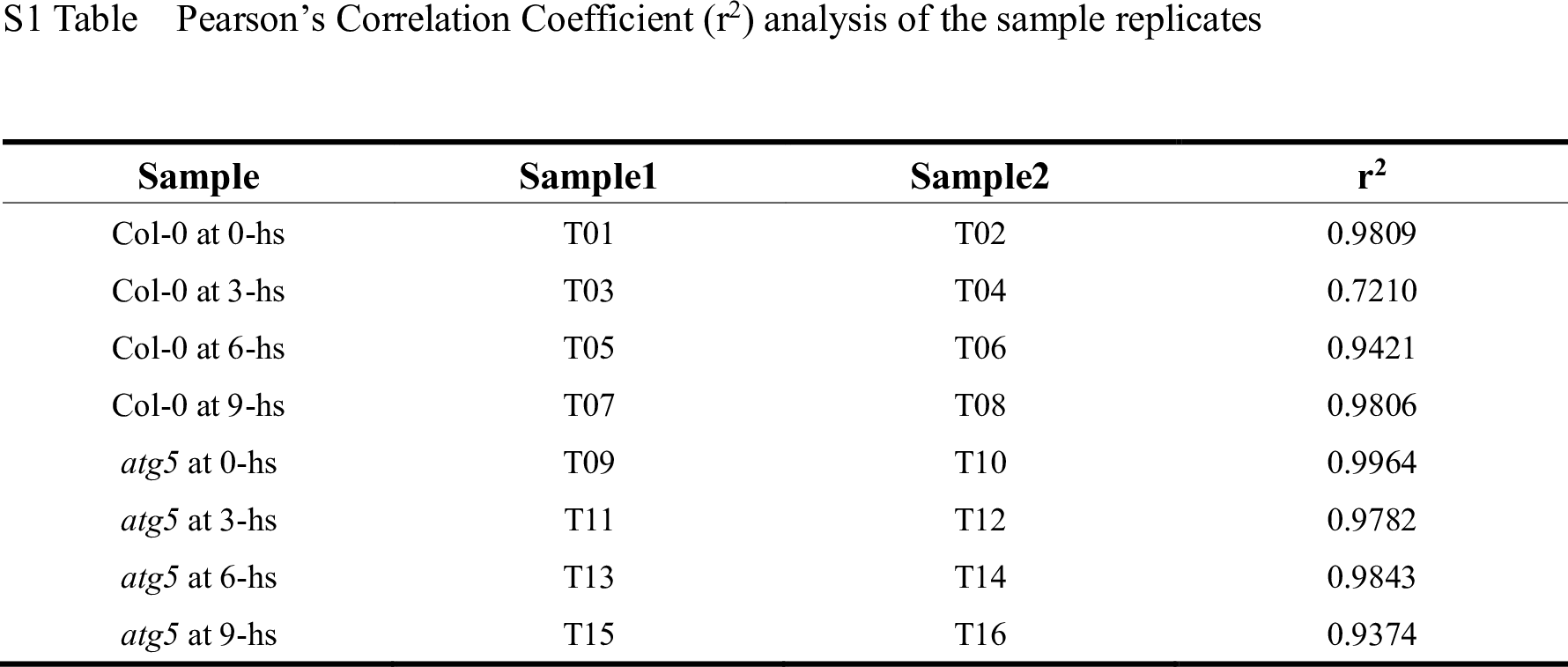

Supplement: S1 Table — (TIF) [file pone.0247783.s007.tif]

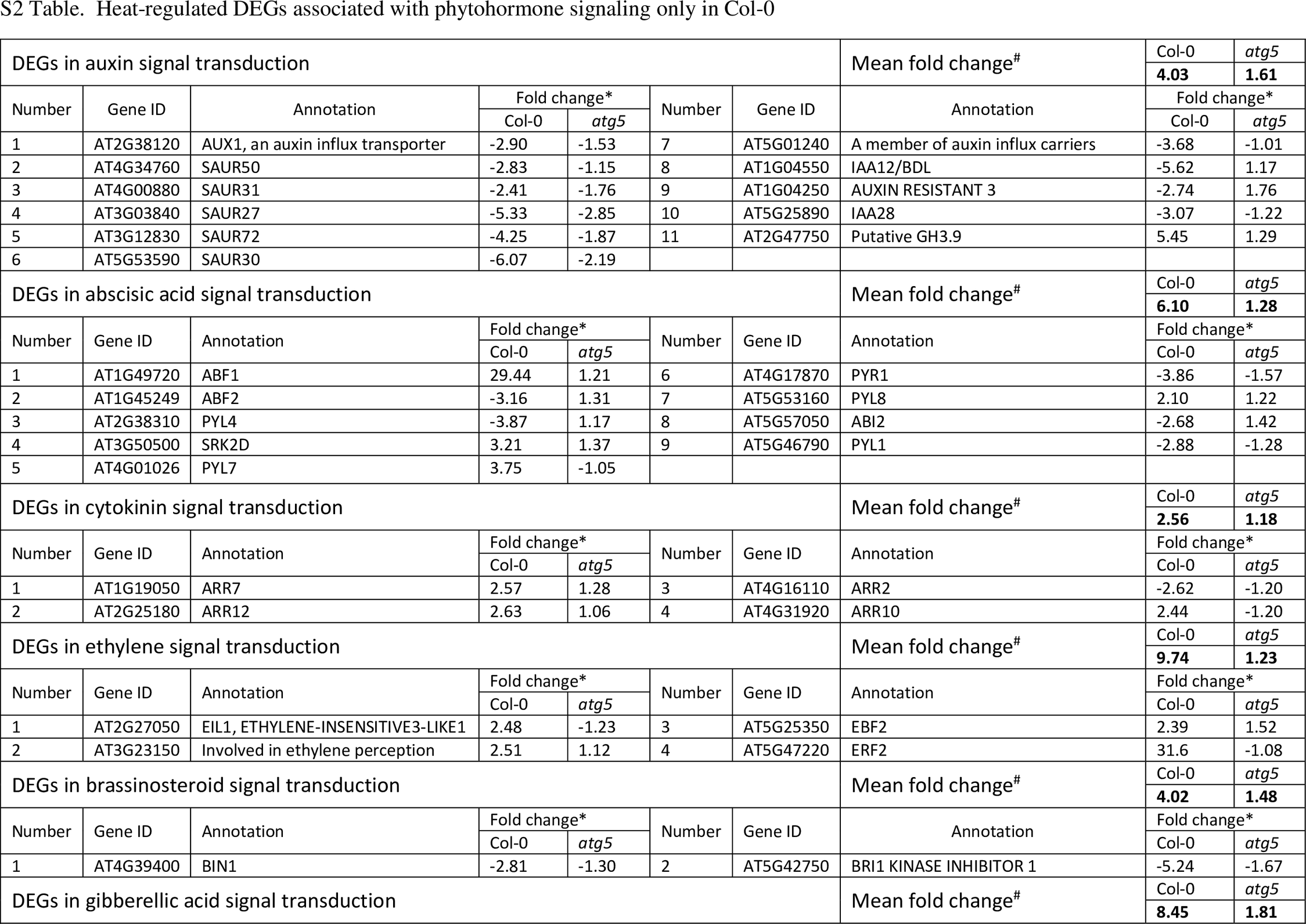

Supplement: S2 Table — (TIF) [file pone.0247783.s008.tif]

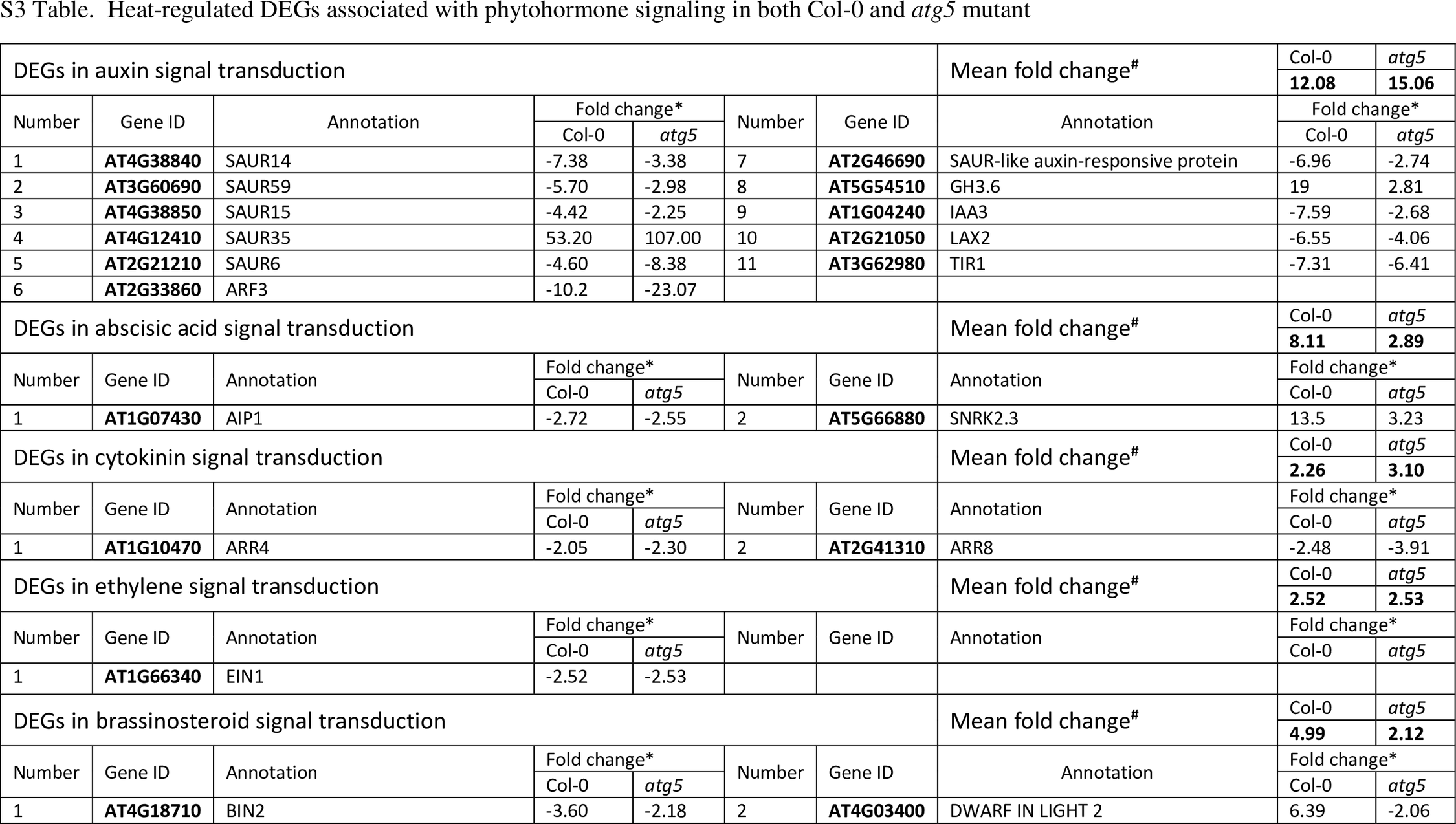

Supplement: S3 Table — (TIF) [file pone.0247783.s009.tif]

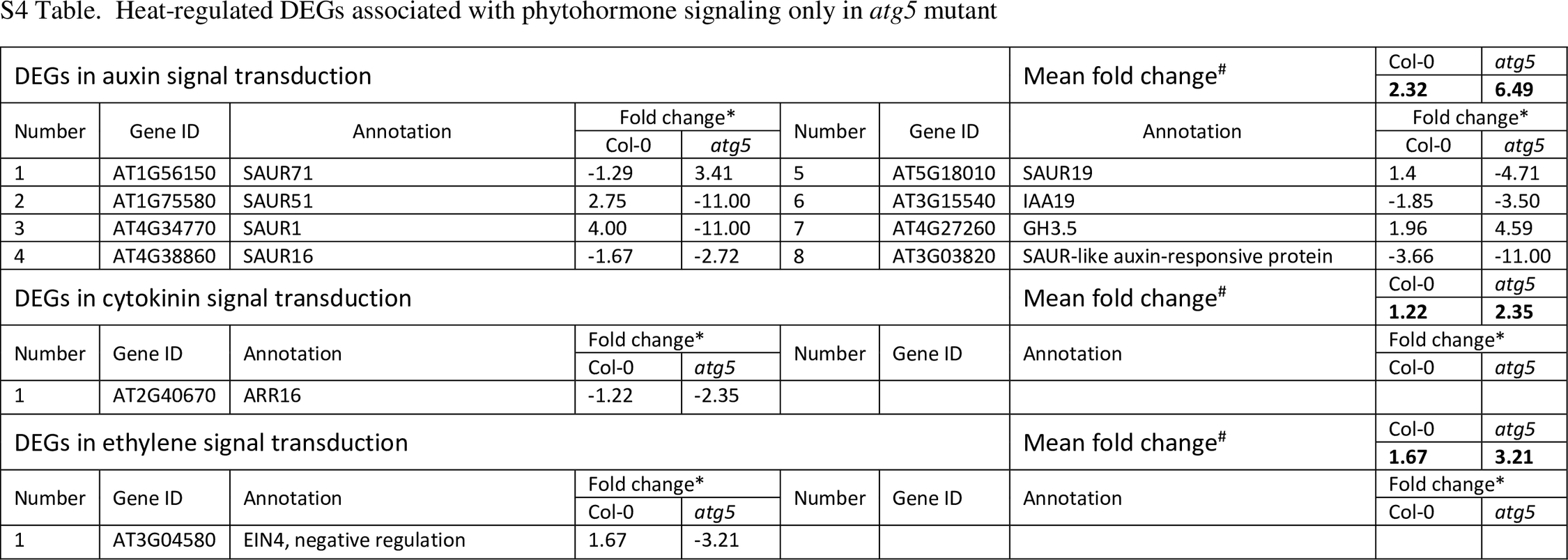

Supplement: S4 Table — (TIF) [file pone.0247783.s010.tif]
